# Supplementary material for: Microenvironment-Mediated Mechanisms of Resistance to HER2 Inhibitors Differ between HER2+ Breast Cancer Subtypes
Source: Cell Syst. Author manuscript; Available in PMC 2018 Apr 30. (PMC5927625; doi:10.1016/j.cels.2018.02.001)
Supplement: 1 [file NIHMS957758-supplement-1.pdf]

Cell Systems, Volume *6*

Supplemental Information

Microenvironment-Mediated Mechanisms of Resistance to HER2 Inhibitors Differer between HER2+ Breast Cancer Subtypes

Spencer S. Watson, Mark Dane, Koei Chin, Zuzana Tatarova, Moqing Liu, Tiera Liby, Wallace Thompson, Rebecca Smith, Michel Nederlof, Elmar Bucher, David Kilburn, Matthew Whitman, Damir Sudar, Gordon B. Mills, Laura M. Heiser, Oliver Jonas, Joe W. Gray, and James E. Korkola

Supplemental Figure S1

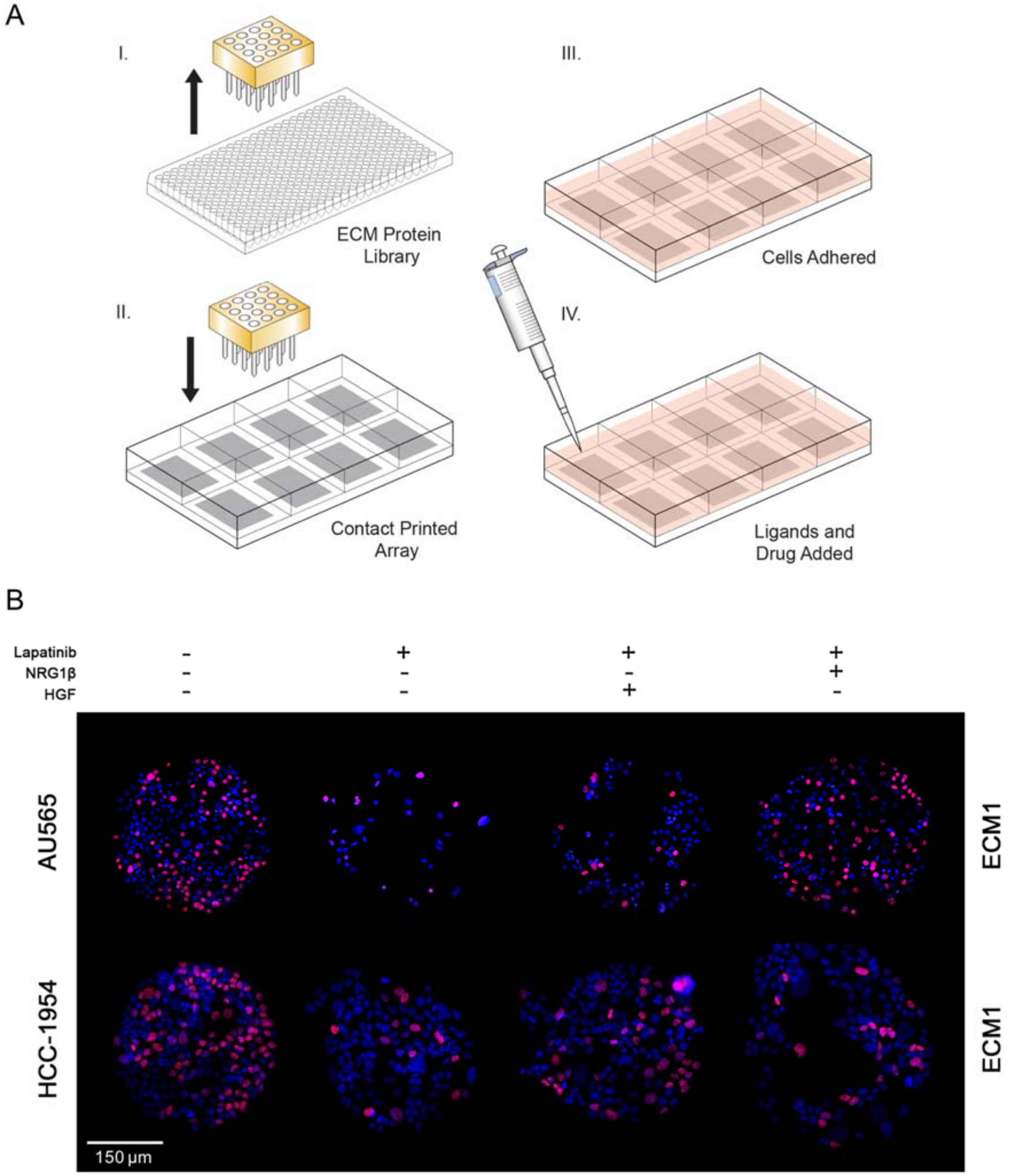

Supplemental Figure S1. Related to Figure 1 and STAR Methods. Schematic of MEMA preparation and representative images of cells grown on MEMA spots. (**a**) Libraries of biologically reactive human ECM and cell adhesion proteins immobilized onto a solid surface by randomized robotic contact printing. Cells adhered to printed protein spots, and exposed to a library of soluble functionalized human ligand proteins and 750 nM lapatinib or DMSO control. (**b**) Composite images of AU565 and HCC1954 cells on MEMA spots containing immobilized ECM1 protein, treated with combinations of lapatinib, NRG1β, and HGF. Nuclei labeled with DAPI (blue), proliferation measured by nuclear EdU uptake (pink).

Supplemental Figure S2

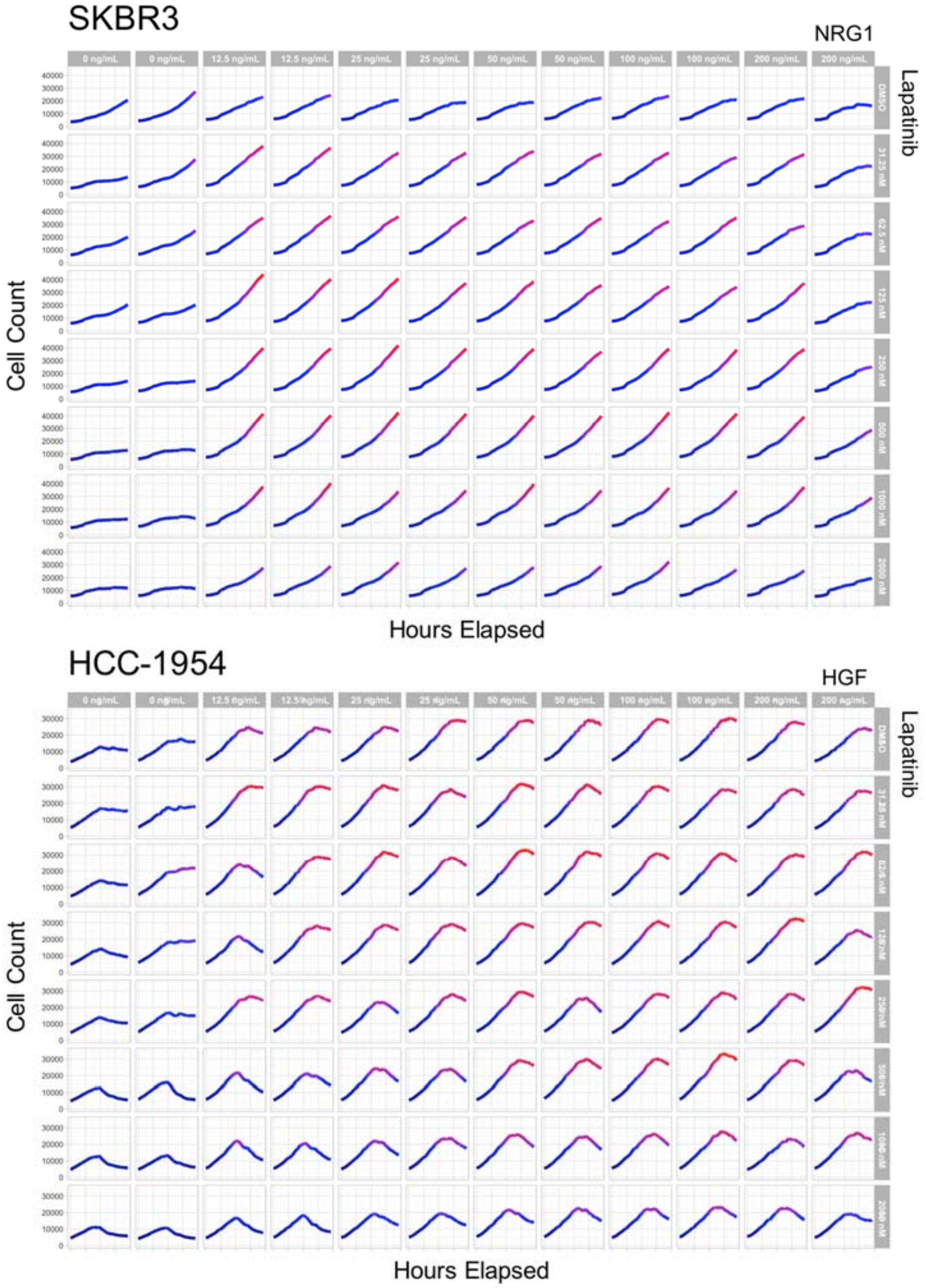

Supplemental Figure S2. Related to Figure 1. Live-cell Imaging reveals a range of concentrations of lapatinib and NRG1β or HGF that either restore proliferation, or enhance it over DMSO treated controls. Line graphs of cell count over 96 hours taken every 2 hours for a dose range of lapatinib versus a concentration range of NRG1β (for SKBR3), or HGF (for HCC1954). Concentration range for both ligands: 12.5, 25, 50, 100, and 200 ng/mL. Cohorts exposed to NRG1β or HGF had ligand added from the start of the experiment. For drug treated cohorts in both cell lines lapatinib was added 24 hours from the start of the experiment. Concentration range for lapatinib: 31.25, 62.5, 125, 250, 500, 1000, and 2000 nM. Red indicates cell count in excess of untreated controls.

Supplemental Figure S3

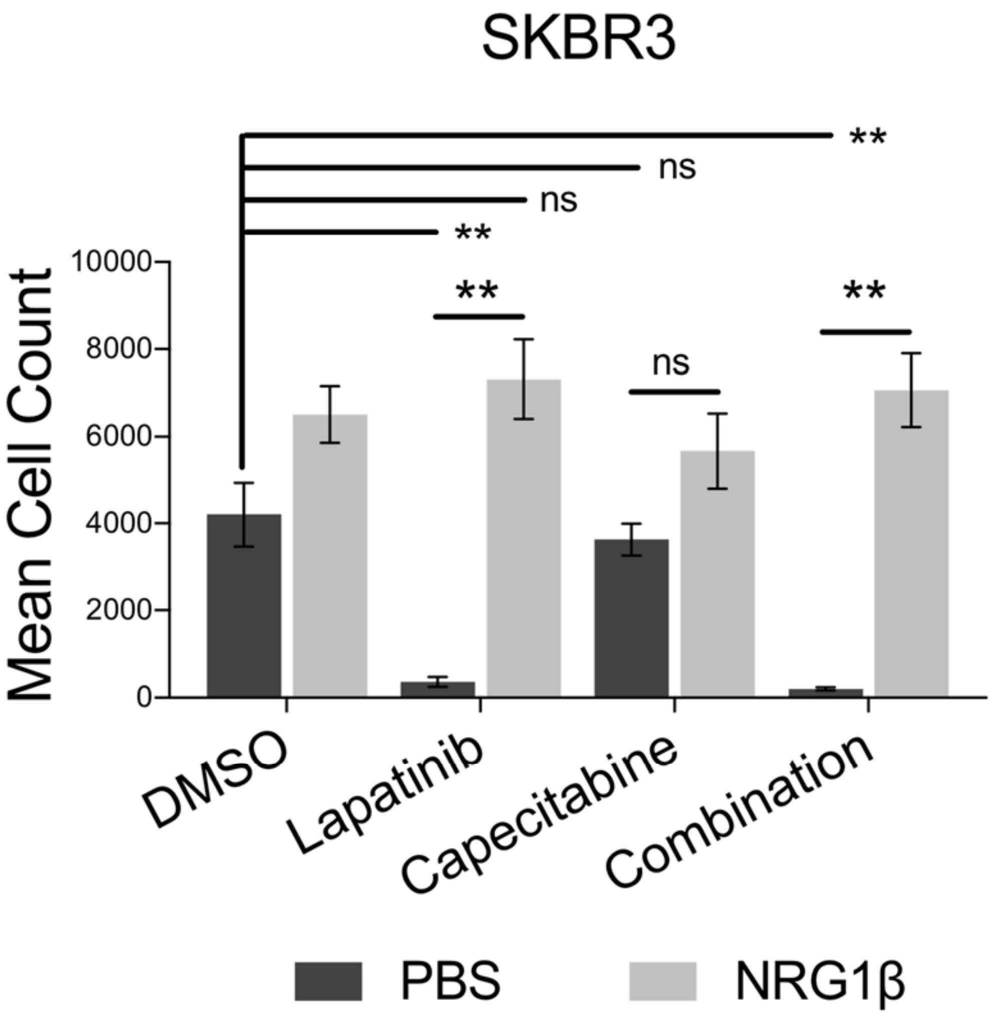

Supplemental Figure S3. Related to Figure 1. NRG1β drives resistance to combination treatment with lapatinib and capecitabine. Mean cell count and SEM (n = 3) for SKBR3 cells treated for 72 hours with combinations of DMSO, 500 nM lapatinib, 500 uM capecitabine, and 50 ng/mL NRG1β or HGF. Lapatinib significantly decreased cell counts compared to DMSO (p=0.0067), and addition of ligand significantly increased cell count compared to lapatinib alone (p=0.0016). Capecitabine decreased cell count compared to DMSO (ns.), but addition of ligand did not significantly increase cell count compared to capecitabine alone. The combination of lapatinib and capecitabine significantly decreased cell counts compared to DMSO (p=0.0056), and the addition of ligand significantly increased cell counts compared to combination (p=0.0012). Lapatinib plus ligand resulted in increased cell count compared to DMSO (ns). DMSO concentrations were matched to capecitabine concentrations.

Supplemental Figure S4

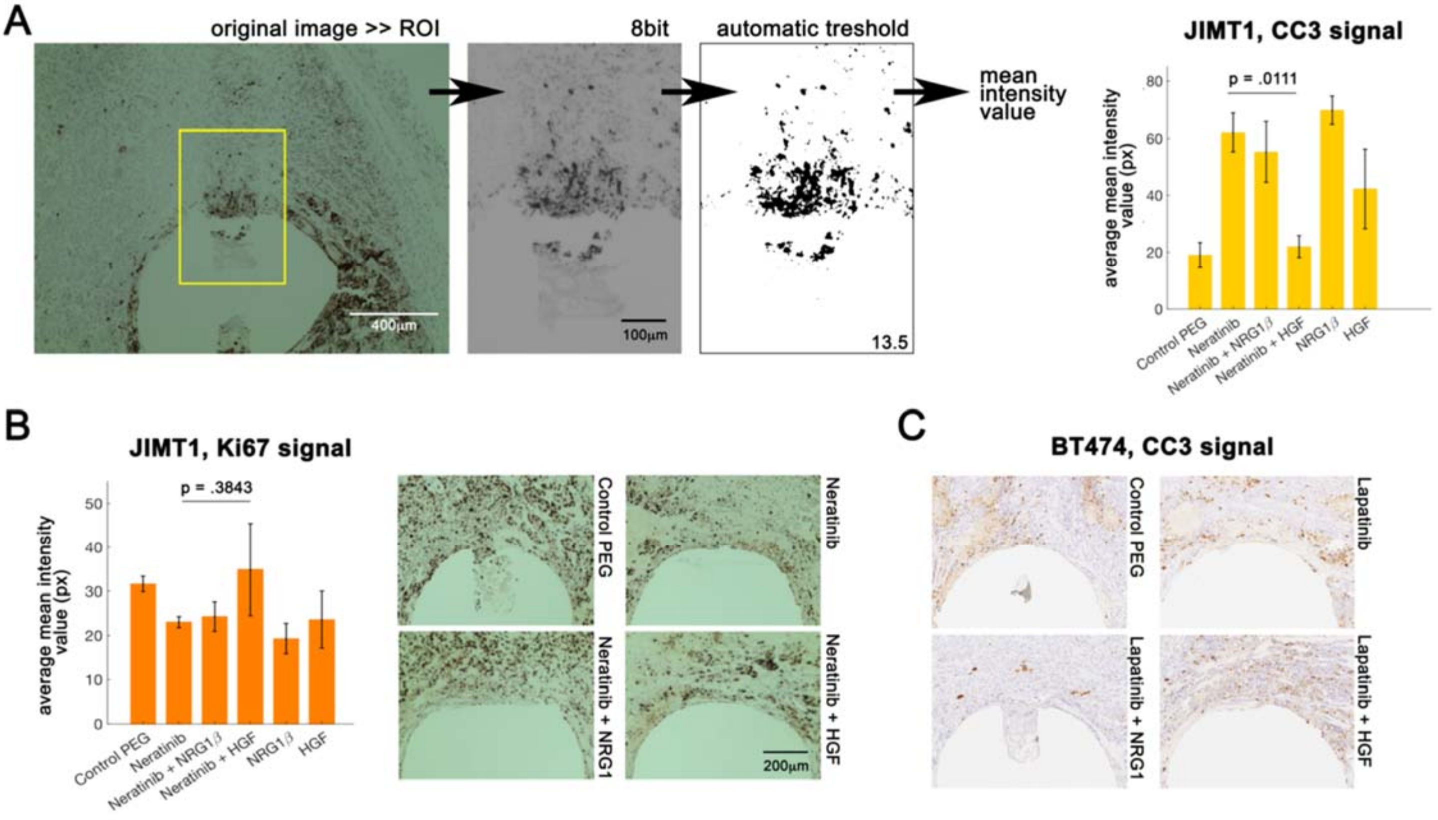

Supplemental Figure S4. Related to Figure 3. NRG1β and HGF mediated resistance to HER2 inhibition in vivo. (**a**) Schematic overview of the semiautomatic signal analysis using ImageJ (Schindelin et al., 2012) Briefly, region of interest was selected with the center at the drug release point. Cropped image was transformed to 8bit and automatic thresholding was applied. Mean intensity was measured and averaged to generate the graphs (right). (**b**) Addition of HGF to Neratinib restored the proliferation to control levels, however, the differences were not significant as compared to the single agent. Quantification (left) and supporting images of Ki67 stained JIMT1 tumor sections in close proximity to the reservoirs are shown (right). (**c**) Basal levels of apoptosis were minor in BT474 xenograft model and thus the effect of different conditions was compared by evaluating the Ki67 signal only (Figure 3E). Values in the graph are means and SEM (n = 3); significance was calculated by a paired sample two-tailed t-test.

Supplemental Figure S5

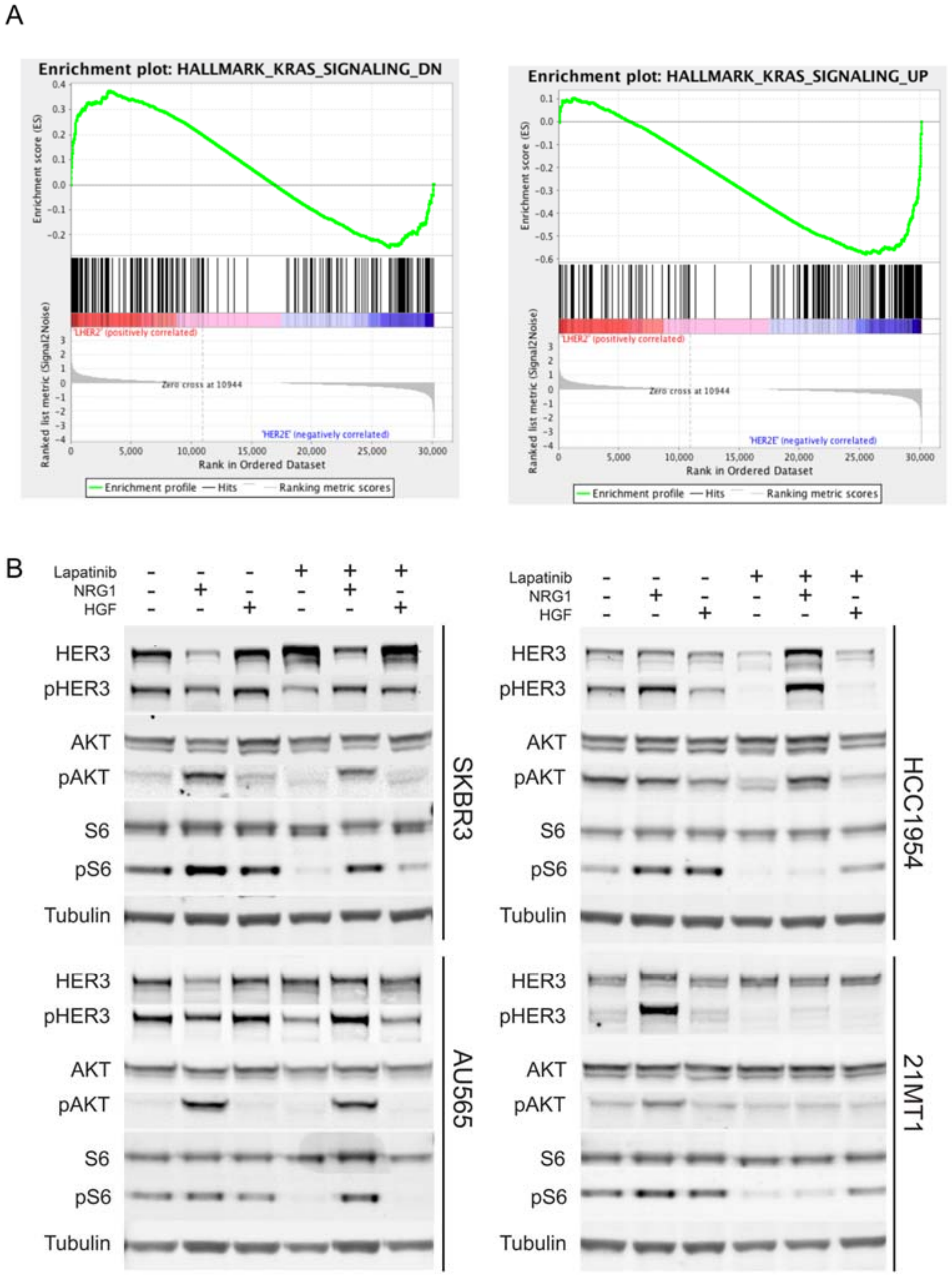

Supplemental Figure S5. Related to Figure 4. L-HER2+ and HER2E have differential MAPK pathway activity and response to HER2 inhibition. (**a**) GSEA comparison plots of L-HER2+ (n = 8) and HER2E lines (n = 8). Top plot shows Hallmark gene set KRAS Down enrichment for L-HER2 vs HER2E (NOM p-val = .003, FDR q-val = .098), and bottom shows KRAS Up gene set enrichment for the reverse comparison (NOM p-val = 0, FDR q-val = 0). (**b**) Western blot protein analysis of AU565, SKBR3, HCC1954, and 21MT1 cell lines treated for 48 hours with combinations of DMSO, 500 nM lapatinib or neratinib, and 50 ng/ml NRG1β or HGF (BR = 3). 21MT1 cells were treated with neratinib due to innate lapatinib resistance.

Supplemental Figure S6

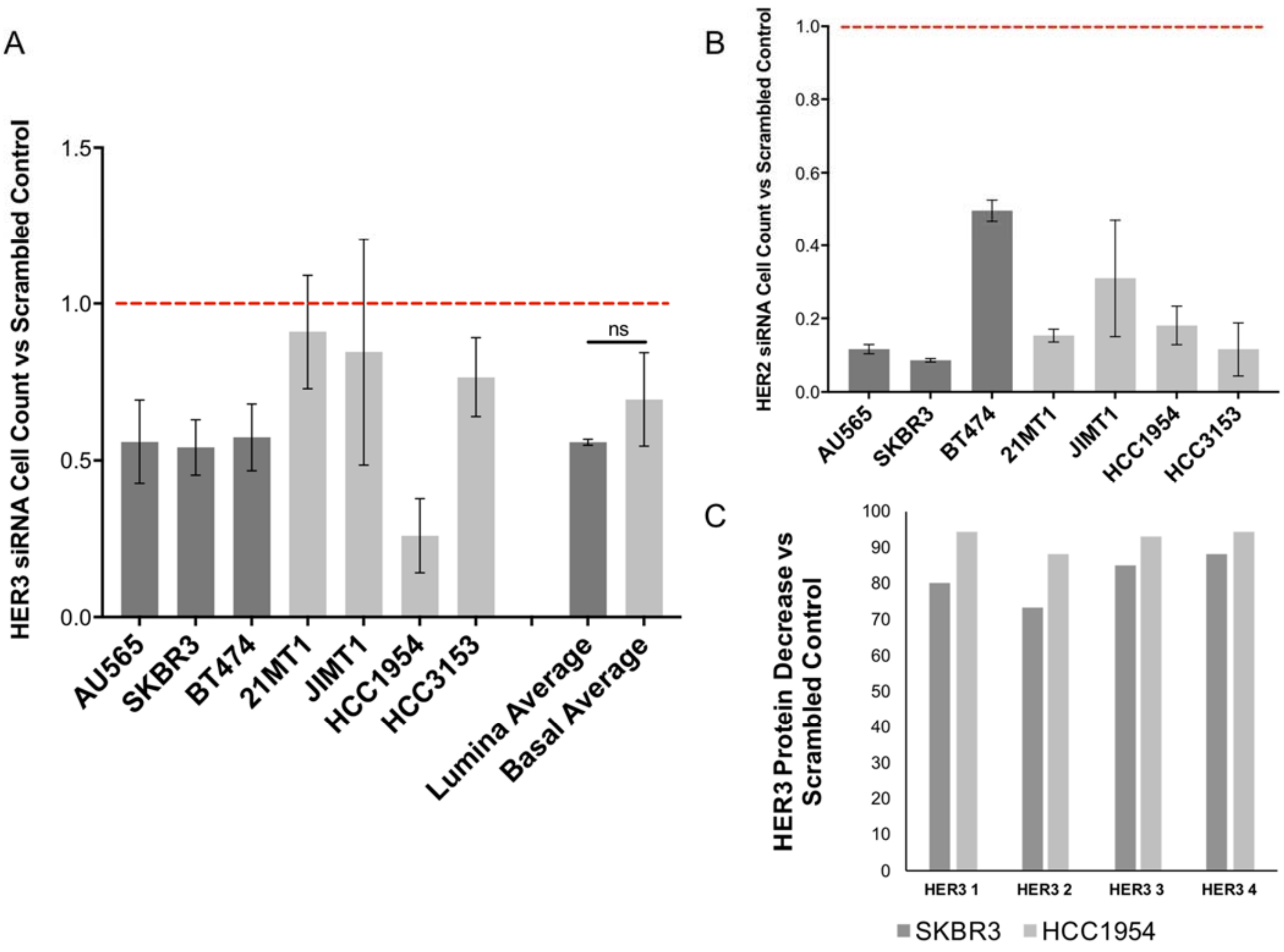

Supplemental Figure S6. Related to Figure 4. HER2E cell lines are less sensitive to HER3 siRNA knockdown than L-HER2+ lines. (**a**) Ratio of mean cell count and SEM (n = 3) of cell lines following 96 hours of HER3 knockdown (averaged over 4 independent HER3 siRNA oligos), normalized to cohorts treated with scrambled siRNA control. Red line indicates equivalent cell counts compared to control. (**b**) Mean cell count and SEM (n = 3) of cell lines following 96 hours of HER2 knockdown, normalized to cohorts treated with scrambled siRNA control. Red line indicates baseline cell count. (**c**) Quantification of HER3 protein immunoblots showing percent decrease in protein levels in SKBR3 and HCC1954 cell lysate following 48 hours of HER3 siRNA knockdown compared to scrambled siRNA controls (BR = 3).

Supplemental Figure S7

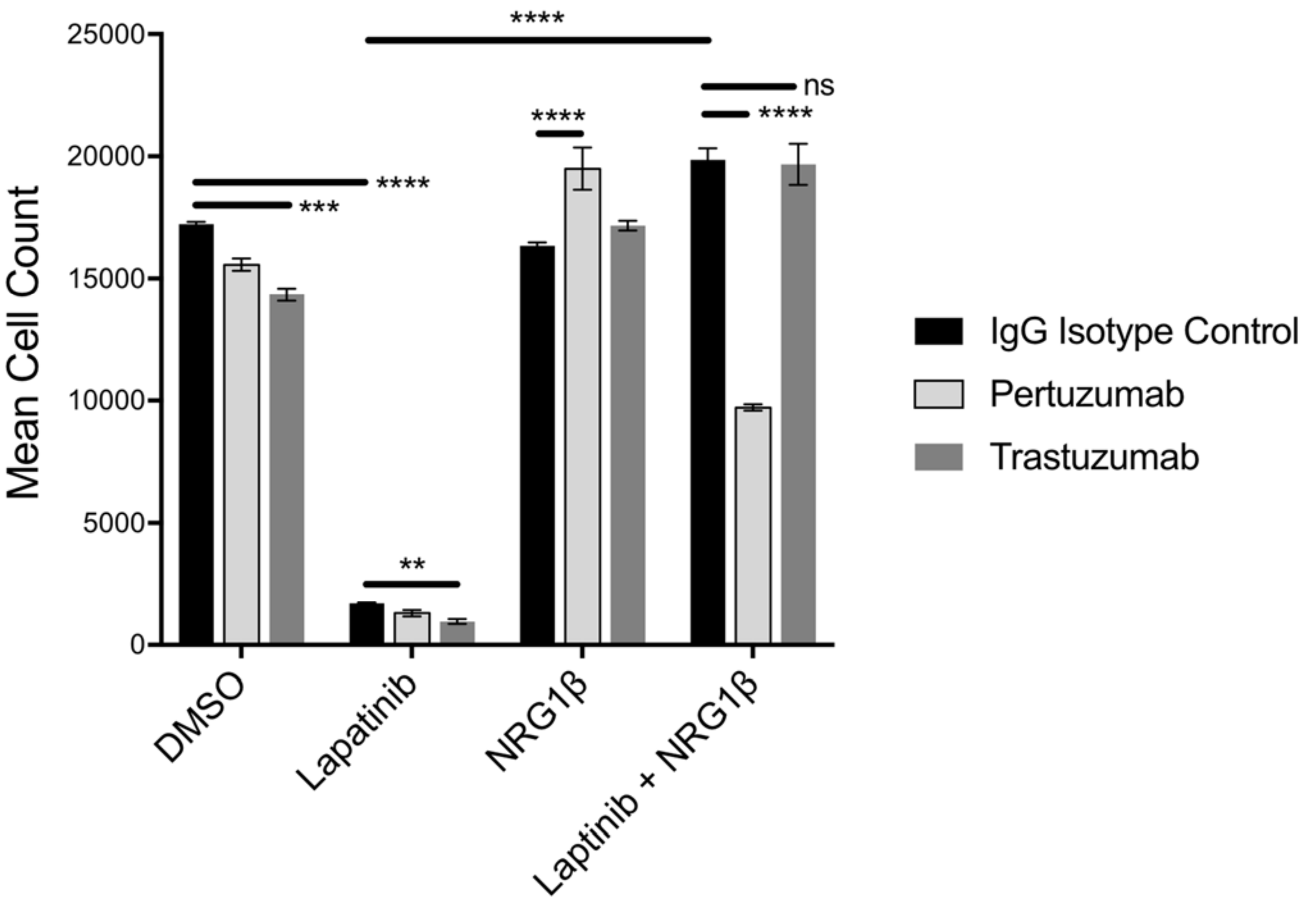

Supplemental Figure S7. Related to Figure 5. Trastuzumab fails to inhibit NRG1β mediated lapatinib resistance. Mean cell count and SEM (n = 3) for AU565 cells treated for 72 hours with combinations of 500 nM lapatinib, 50 ng/mL NRG1β, 30 µg/mL pertuzumab, and 30 µg/mL trastuzumab. PBS, DMSO, and human IgG isotype control were used as controls for growth factors, lapatinib, and pertuzumab or trastuzumb, respectively. Lapatinib significantly decreased cell counts compared to DMSO ($p<0.0001$), and addition of ligand significantly increased cell count compared to lapatinib alone ($p<0.0001$)). Trastuzumab significantly decreased cell count compared to DMSO ($p=0.0005$). The combination of pertuzumab and NRG1β significantly increased cell count compared to NRG1β alone ($p<0.0001$). Addition of pertuzumab to lapatinib plus NRG1β significantly decreased cell counts compared to lapatinib plus NRG1β alone ($p<0.0001$), but addition of trastuzumab to lapatinib plus NRG1β did not.
