# Supplementary material for: Microenvironment-Mediated Mechanisms of Resistance to HER2 Inhibitors Differ between HER2+ Breast Cancer Subtypes
Source: Cell Syst. Author manuscript; Available in PMC 2018 Apr 30. (PMC5927625; doi:10.1016/j.cels.2018.02.001)
Supplement: 2 [file NIHMS957758-supplement-2.pdf]

Western blots from Figure 4B. Imaged on LI-COR Odyssey, 2-channel fluorescence on same blots.

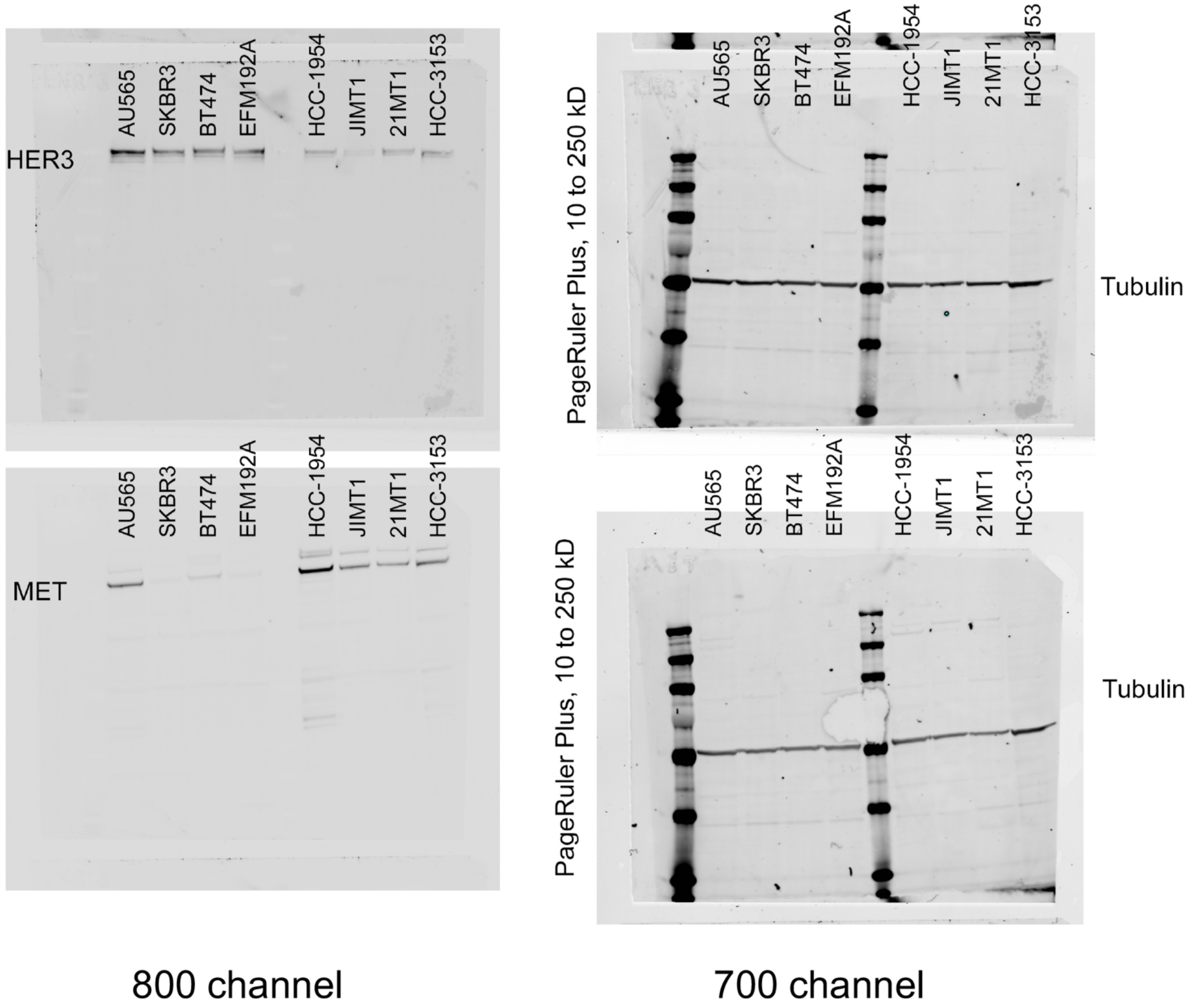

Phospho-protein Western blots from Figure S5B. Imaged on LI-COR Odyssey, 2-channel fluorescence on same blots.

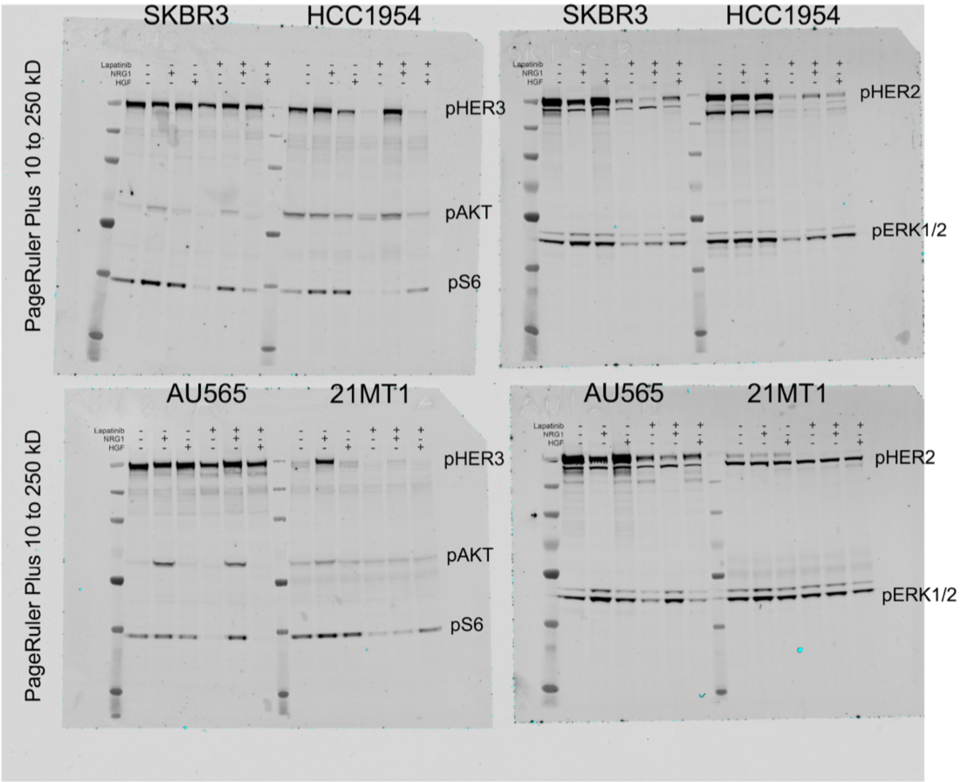

700+800 channel

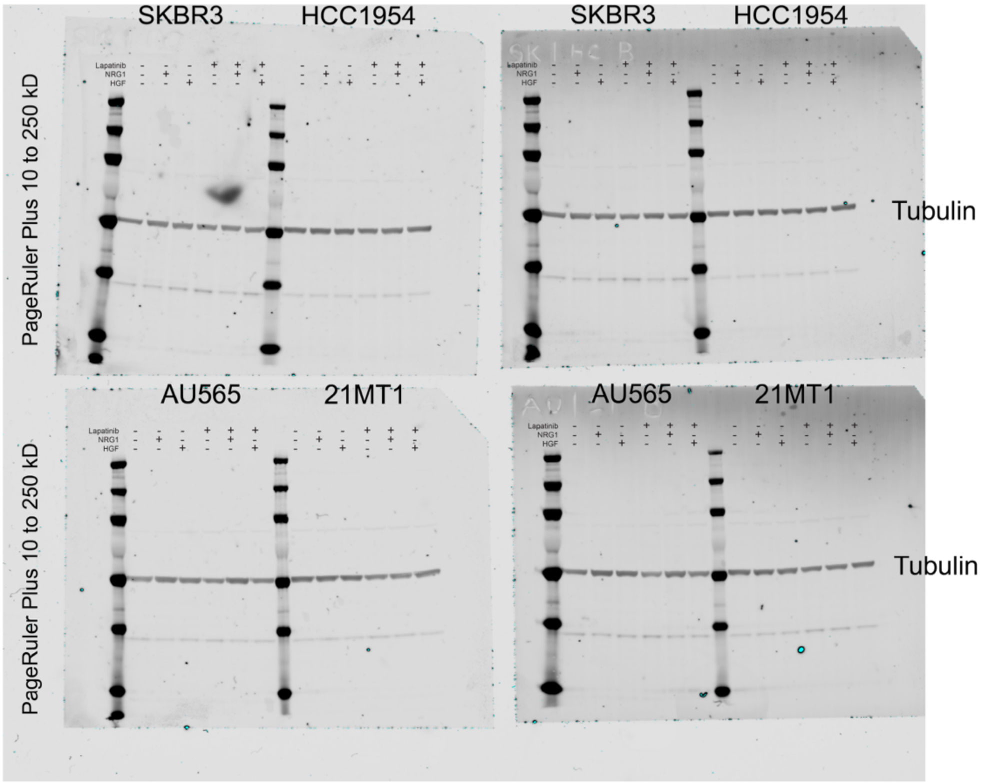

800 channel

Total-protein Western blots from Figure S5B. Imaged on LI-COR Odyssey, 2-channel fluorescence on same blots.

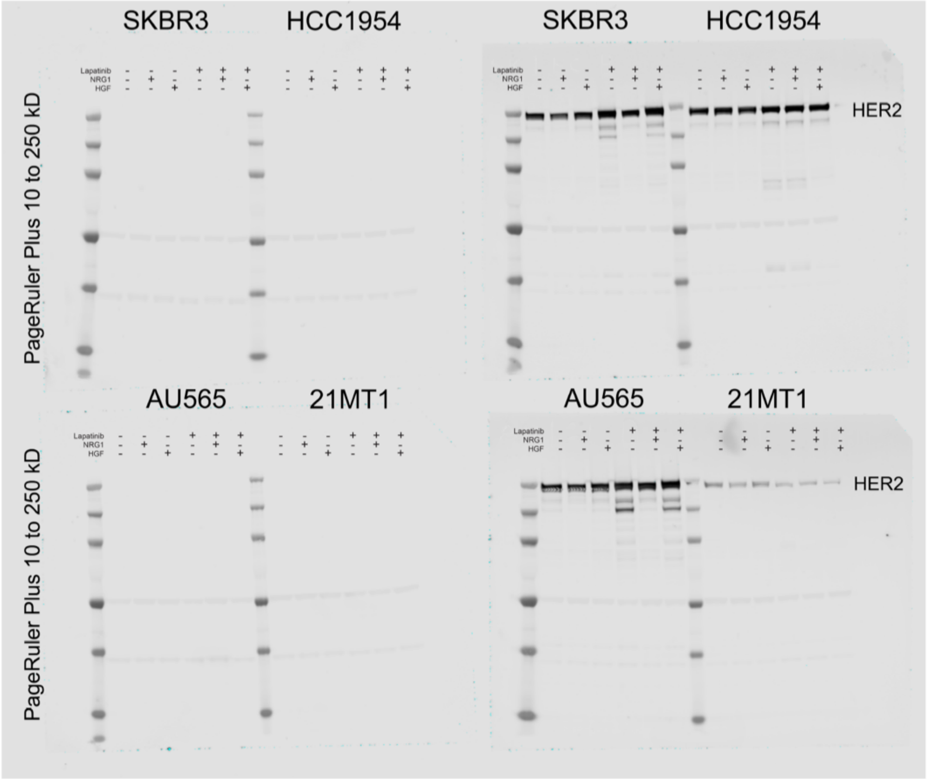

800 channel (low exposure)

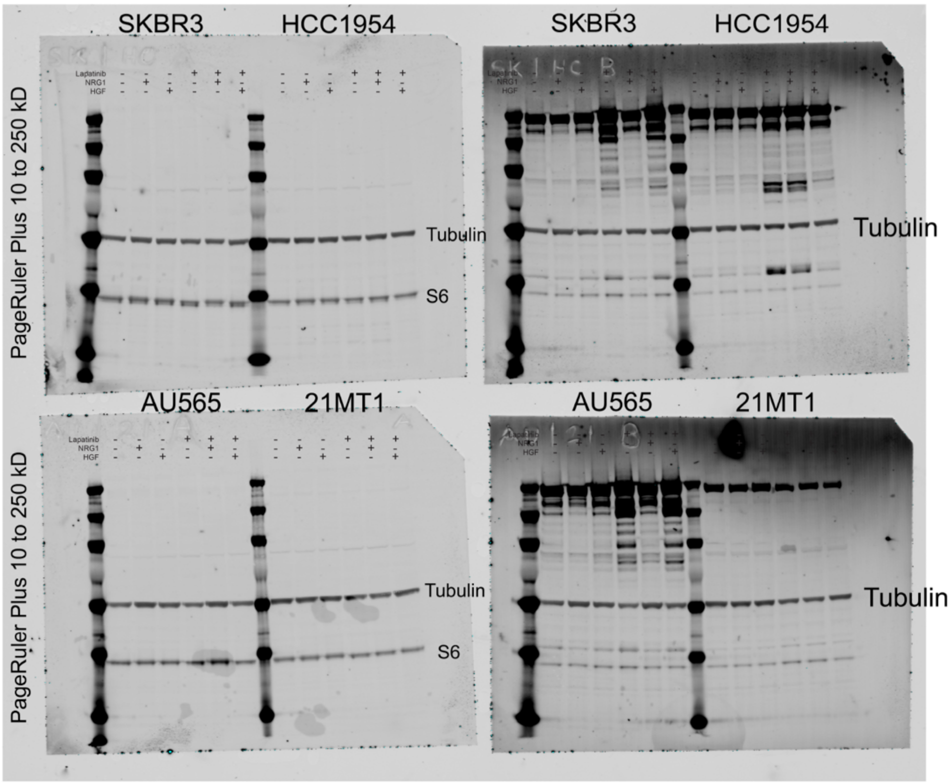

800 channel (high exposure)

Total-protein Western blots from Figure S5B. Imaged on LI-COR Odyssey, 2-channel fluorescence on same blots.

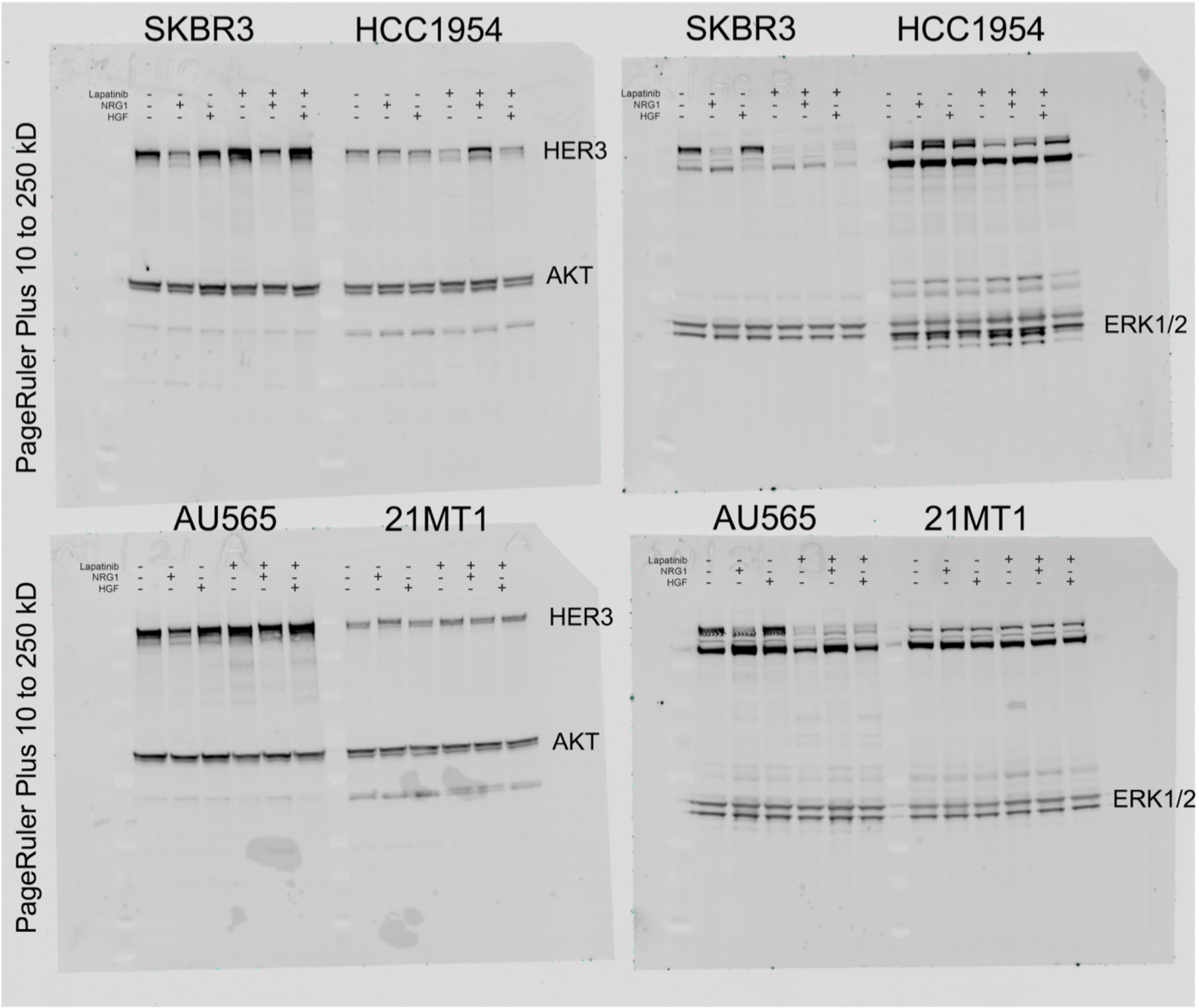

700 channel

Western blots from Supplemental Figure S6B. Imaged on LI-COR Odyssey, 2-channel fluorescence on same blots.

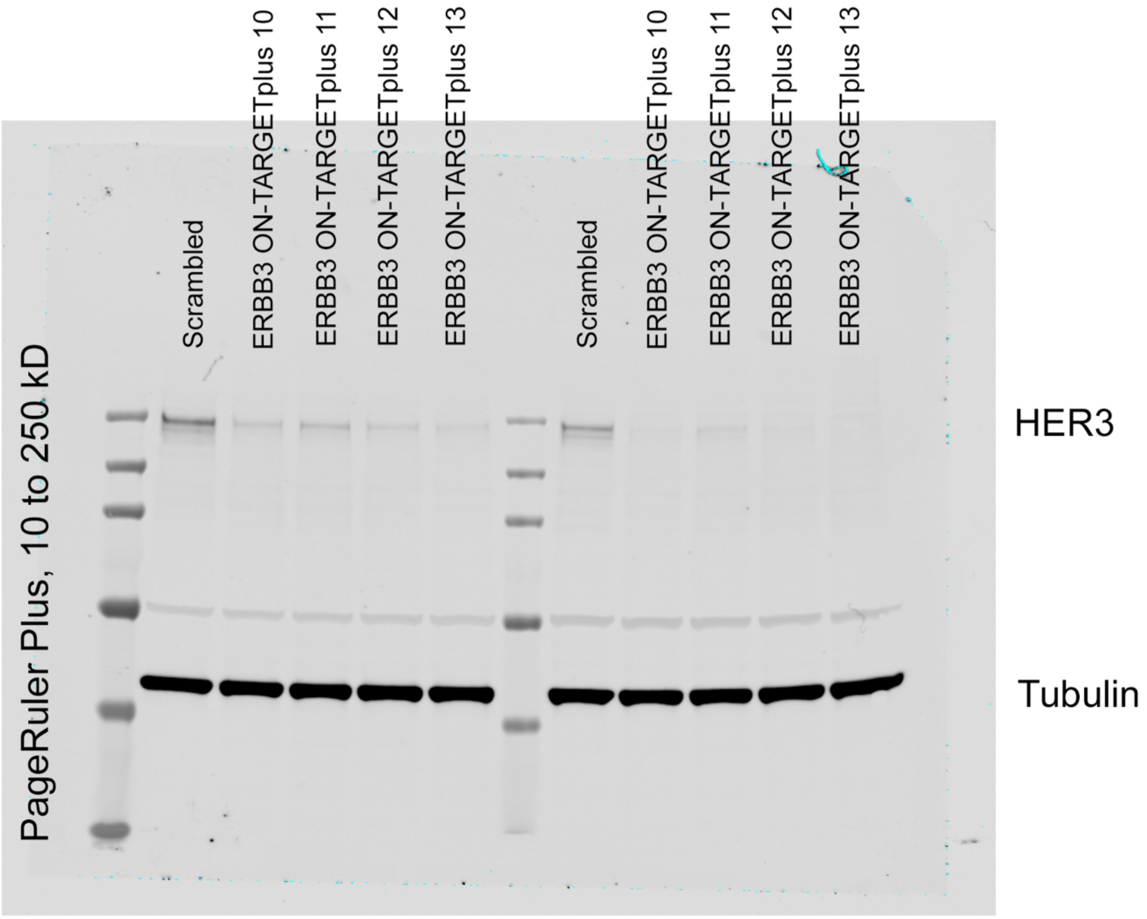

700+800 channel
