# Supplementary material for: Microenvironment-Mediated Mechanisms of Resistance to HER2 Inhibitors Differ between HER2+ Breast Cancer Subtypes
Source: Cell Syst. Author manuscript; Available in PMC 2018 Apr 30. (PMC5927625; doi:10.1016/j.cels.2018.02.001)
Supplement: 3 [file NIHMS957758-supplement-3.pdf]

Cell Systems

Article

Microenvironment-Mediated Mechanisms of Resistance to HER2 Inhibitors Differ between HER2+ Breast Cancer Subtypes

Graphical Abstract

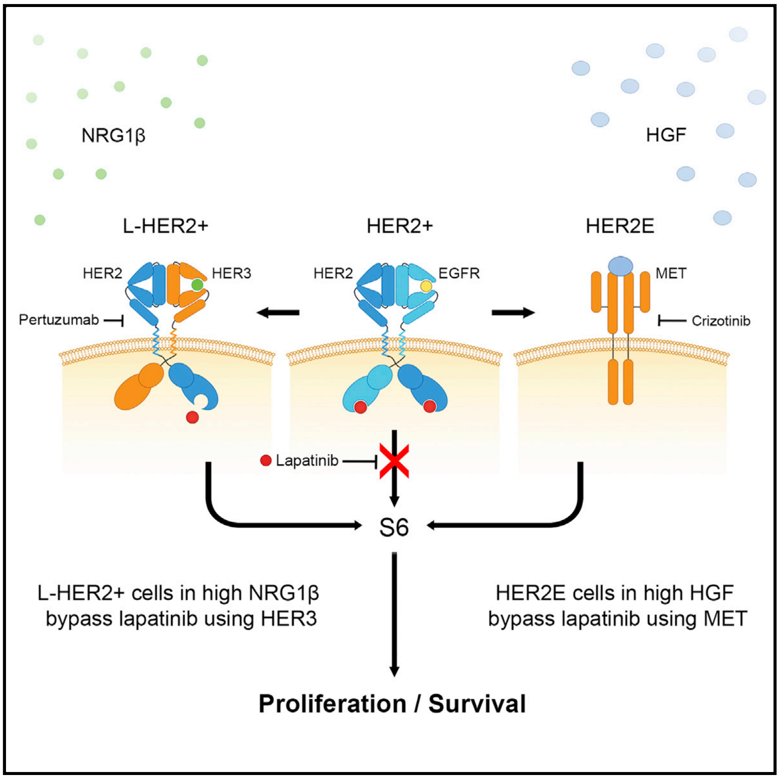

Highlights

- High-throughput assay to assess microenvironment (ME) impacts on drug resistance
- L-HER2+ and HER2E cells differ in TKI sensitivity, pathway use, and ME influence
- NRG1β and HGF reduce TKI efficacy in L-HER2+ and HER2E cells, respectively
- Pertuzumab and crizotinib counter NRG1β- and HGF-induced TKI resistance, respectively

Authors

Spencer S. Watson, Mark Dane, Koei Chin, ..., Oliver Jonas, Joe W. Gray, James E. Korkola

Correspondence

grayjo@ohsu.edu (J.W.G.), korkola@ohsu.edu (J.E.K.)

In Brief

We describe a powerful platform for discovery of microenvironment signals that influence drug responses. We show through application of the platform to HER2+ breast cancer cell lines that NRG1β and HGF suppress responses to lapatinib and neratinib in L-HER2+ and HER2E cells, respectively. We show that these differences are caused by differences in epigenomic status and regulatory pathway use between L-HER2+ and HER2E breast cancers. We also present evidence suggesting that microenvironment-mediated resistance to HER2-targeted tyrosine kinase inhibitors can be overcome in L-HER2+ cancers by co-treatment with pertuzumab, and in HER2E cancers by co-treatment with crizotinib or trametinib.

Watson et al., 2018, Cell Systems 6, 329–342
March 28, 2018 © 2018 The Authors. Published by Elsevier Inc.
https://doi.org/10.1016/j.cels.2018.02.001

Cell Systems
Article

OPEN ACCESS
CellPress

Microenvironment-Mediated Mechanisms of Resistance to HER2 Inhibitors Differ between HER2+ Breast Cancer Subtypes

Spencer S. Watson,[1] Mark Dane,[1] Koei Chin,[1] Zuzana Tatarova,[1] Moqing Liu,[1] Tiera Liby,[1] Wallace Thompson,[1] Rebecca Smith,[1] Michel Nederlof,[1,4] Elmar Bucher,[1] David Kilburn,[1] Matthew Whitman,[3] Damir Sudar,[1,4] Gordon B. Mills,[2] Laura M. Heiser,[1] Oliver Jonas,[3] Joe W. Gray,[1,*] and James E. Korkola[1,5,*]

[1]Department of Biomedical Engineering, Knight Cancer Institute, OHSU Center for Spatial Systems Biomedicine, Oregon Health and Science University, 3181 SW Sam Jackson Park Road, Portland, OR 97239, USA

[2]Department of Systems Biology, The University of Texas MD Anderson Cancer Center, 1515 Holcombe Boulevard, Houston, TX 77030, USA

[3]Department of Radiology, Brigham & Women's Hospital, Harvard Medical School, 75 Francis St, Boston, MA 02115, USA

[4]Quantitative Imaging Systems LLC, 1410 NW Kearney Street, #1114, Portland, OR 97209, USA

[5]Lead Contact

*Correspondence: grayjo@ohsu.edu (J.W.G.), korkola@ohsu.edu (J.E.K.)

https://doi.org/10.1016/j.cels.2018.02.001

SUMMARY

Extrinsic signals are implicated in breast cancer resistance to HER2-targeted tyrosine kinase inhibitors (TKIs). To examine how microenvironmental signals influence resistance, we monitored TKI-treated breast cancer cell lines grown on microenvironment microarrays composed of printed extracellular matrix proteins supplemented with soluble proteins. We tested ∼2,500 combinations of 56 soluble and 46 matrix microenvironmental proteins on basal-like HER2+ (HER2E) or luminal-like HER2+ (L-HER2+) cells treated with the TKIs lapatinib or neratinib. In HER2E cells, hepatocyte growth factor, a ligand for MET, induced resistance that could be reversed with crizotinib, an inhibitor of MET. In L-HER2+ cells, neuregulin1-β1 (NRG1β), a ligand for HER3, induced resistance that could be reversed with pertuzumab, an inhibitor of HER2-HER3 heterodimerization. The subtype-specific responses were also observed in 3D cultures and murine xenografts. These results, along with bioinformatic pathway analysis and siRNA knockdown experiments, suggest different mechanisms of resistance specific to each HER2+ subtype: MET signaling for HER2E and HER2-HER3 heterodimerization for L-HER2+ cells.

INTRODUCTION

Overexpression of HER2 occurs in ∼25% of all breast cancers due to amplification of the *HER2* locus at 17q12 and is associated with aggressive tumor behavior and poor outcome in the absence of HER2 targeted therapy (Slamon et al., 1989; Sorlie et al., 2003). However, outcomes have been substantially improved with the use of therapeutic agents that target HER2, such as the monoclonal antibody drugs trastuzumab and pertuzumab, and the small-molecule, orally available tyrosine kinase inhibitors (TKIs) lapatinib and neratinib. Clinical studies with HER2-targeted agents have shown improved outcomes over chemotherapy alone for patients with HER2+ breast cancer in both the metastatic and adjuvant settings (Arteaga et al., 2011). Lapatinib was approved by the US Food and Drug Administration (FDA) for the treatment of HER2+ breast cancer in combination with letrozole (Johnston et al., 2009) or capecitabine (Geyer et al., 2006) and shows promise when combined with trastuzumab (de Azambuja et al., 2014). Neratinib has recently been approved by the FDA for extended adjuvant treatment of early-stage HER2+ breast cancer (Tiwari et al., 2016). However, responses to these TKIs vary between patients (Gomez et al., 2008; Kaufman et al., 2009) and in advanced cancers are usually not durable (Dieras et al., 2017).

Multiple resistance mechanisms have been proposed, but most studies have focused on intrinsic properties of the tumor cells themselves. We sought to determine how both soluble factors and extracellular matrix (ECM) proteins from the microenvironment affect response to the HER2-targeted TKIs lapatinib or neratinib. We were motivated by several recent studies that have demonstrated that extrinsic signals from the tumor microenvironment allow otherwise drug-sensitive cancer cells to escape therapeutic control. Paracrine growth factors (Wilson et al., 2012; DeNardo et al., 2011), ECM proteins, and physical structure (Huang et al., 2011; Acerbi et al., 2015; Muranen et al., 2012) and hypoxia (Sullivan et al., 2008) all have been implicated in breast cancer drug resistance.

We used an emerging technology, microenvironment microarrays (MEMA) (Lin et al., 2012) to study microenvironment effects on anti-HER2 TKI response. MEMA consist of functional proteins printed into well plates to form pads upon which cells grow. We added soluble ligands to each well, allowing us to assess the effects of thousands of unique combinatorial microenvironments on cell response. We found that both soluble and ECM factors from diverse microenvironments diminished responses to the HER2-targeted TKIs. We also showed that the factors conferring resistance differed between luminal-like (L-HER2+) and basal-like (HER2E) HER2+ subtypes as defined

Check for updates

This is an open access article under the CC BY license (http://creativecommons.org/licenses/by/4.0/).

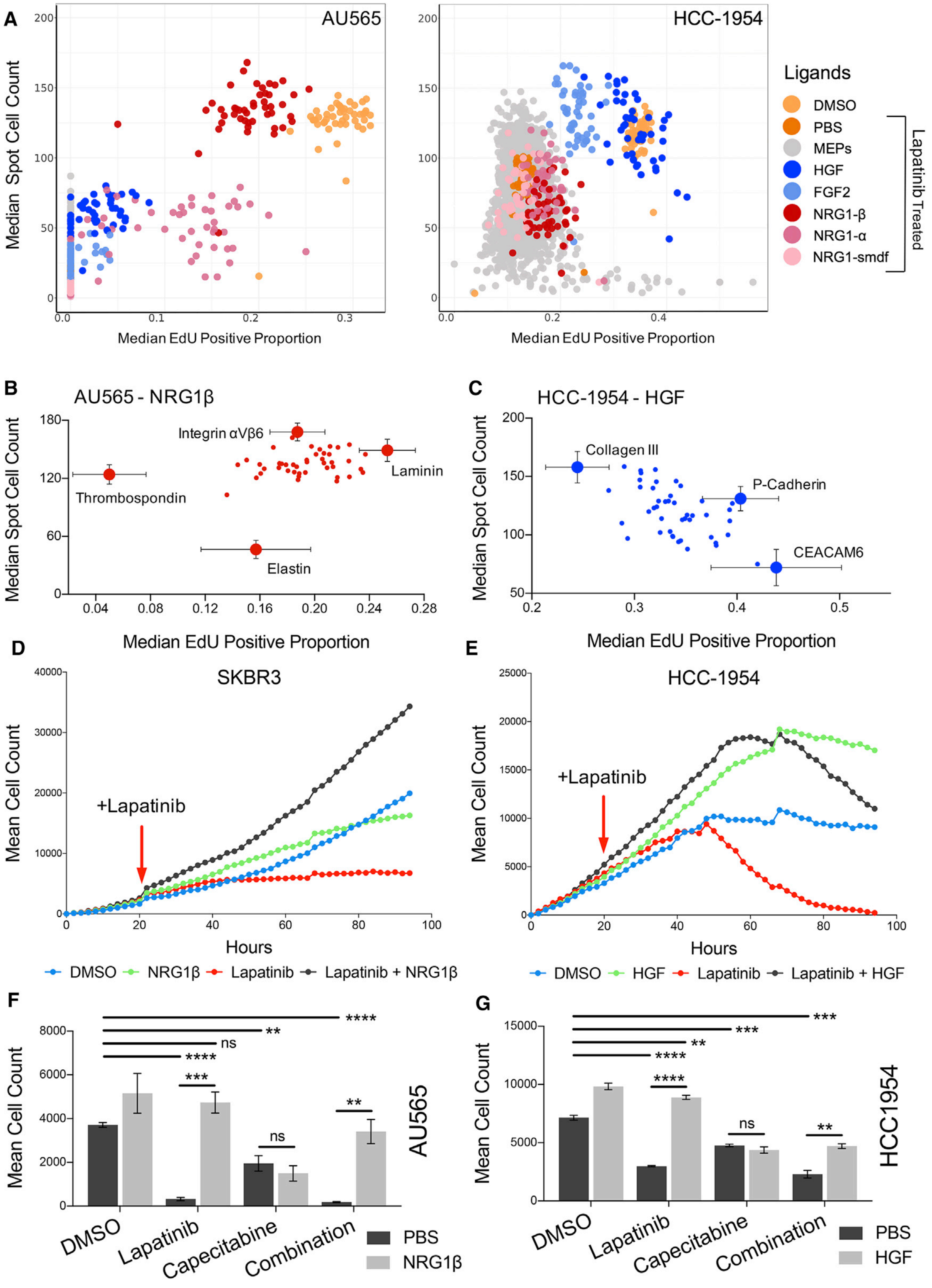

Figure 1. MEMA Studies Reveal Multiple Protein Combinations that Confer Lapatinib Resistance to Otherwise Sensitive HER2+ Breast Cancer Cell Lines

(A) Plots of median cell count versus median EdU-positive ratio for AU565 and HCC1954 cells on MEMAs following 72 hr of 750 nM lapatinib or DMSO treatments. Microenvironment pertubagens (MEPs, combination of ECM and ligand) are color coded by ligand and treatment. Non-EGF, FGF, and HGF family ligands are shown in gray.

(legend continued on next page)

by the TCGA (Cancer Genome Atlas Network, 2012). Neuregulin1-β1 (NRG1β) conferred resistance to L-HER2+ subtype cells, and hepatocyte growth factor (HGF) conferred resistance in HER2E cells, but not vice versa. These differential responses to microenvironmental factors reflect fundamental differences in signaling network wiring and architecture in the two subtypes. The microenvironment-mediated resistance was reversed by co-treatment with pertuzumab in L-HER2+ cells and by co-treatment with crizotinib in HER2E cells. Our findings also support the emerging notion that L-HER2+ and HER2E represent distinct diseases. They also suggest clinical studies to test the possibility that differential targeting of resistance factors from the microenvironment in L-HER2+ and HER2E will improve clinical outcome in patients being treated with HER2-targeted TKIs.

RESULTS

Microenvironment Microarrays Identify Factors Causing Resistance to Lapatinib

Our initial studies used MEMAs (Lin et al., 2012) to identify specific soluble and matrix microenvironmental proteins that altered response to lapatinib in HER2+ cell lines. We grew either AU565 cells (representing the L-HER2+ subtype) or HCC1954 cells (representing the HER2E subtype) on comprehensive MEMA sets under lapatinib treatment or control conditions (STAR Methods, Figure S1A). Growth of the cells on MEMA allowed assessment of the effects of >2,500 different combinations of 56 soluble and 46 matrix microenvironment proteins on drug response. We fixed, stained, and imaged the arrays, then quantified the images using Cell Profiler software (STAR Methods, Figures S1A and S1B). Data from 256 arrays were normalized by RUV and LOESS regression to reduce variation in cell counts and staining intensity (Gagnon-Bartsch et al., 2013).

We assessed the impacts of the diverse microenvironments after 72 hr of growth on cell count and/or the fraction of cells incorporating 5-ethynyl-2′-deoxyuridine (EdU) after lapatinib treatment compared with DMSO control. The full control and treatment data for both AU565 and HCC1954 are available at (http://lincsportal.ccs.miami.edu/datasets-beta/#?query=assayname:MEMA cell growth assay). Figures 1A and 1B show that several soluble and matrix factors influenced cell growth and EdU incorporation during treatment with lapatinib. NRG1 isoforms attenuated response to lapatinib in AU565 (Figure 1A), while FGF2 and HGF attenuated response to lapatinib in HCC1954 (Figure 1A) but not vice versa. The degree of attenuation of response in AU565 differed between NRG1 isoforms and other epidermal growth factor (EGF) family members. For example, the post-treatment cell count and fraction of EdU-incorporating cells for AU565 cells treated with lapatinib and NRG1β were similar to those for vehicle control-treated AU565 cells. Treatment with lapatinib and NRG1α resulted in a lower fraction of EdU-incorporating cells compared with NRG1β and DMSO treatments, but a higher average cell count than most lapatinib-treated conditions. The NRG1-SMDF isoform had little effect on cell count or fraction of EdU-incorporating cells under lapatinib treatment.

Although soluble factors had the largest impact on cell response, matrix proteins also influenced drug response to a lesser degree. NRG1β-mediated attenuation of response to lapatinib in AU565 cells was diminished by growth of cells on thrombospondin and elastin and enhanced by growth on laminin and integrin αVβ6 (Figure 1B). HGF attenuation of response to lapatinib in HCC1954 cells was decreased by growth on collagen III and enhanced by growth on P-cadherin and CEACAM6 (Figure 1C). Overall, NRG1β and HGF were the strongest microenvironmental inhibitors of lapatinib response in AU565 and HCC1954, respectively.

We next assessed the effects of a range of NRG1β and HGF concentrations on responses to a range of lapatinib doses in a 2D live-cell assay in SKBR3 and HCC1954 cells expressing nuclear-GFP. Nuclear-GFP SKBR3 cells were used in place of AU565 due to their similar expression profiles and previous validation of their use in live-cell assays (Heiser et al., 2012). The inhibitory effect of lapatinib was previously observed to be cytostatic in L-HER2+ breast cancer cell lines, rather than cytotoxic (Diermeier-Daucher et al., 2011). Thus, we only measured cell count in L-HER2+ cells. All concentrations of NRG1β diminished lapatinib efficacy in SKBR3 cells and several concentrations of NRG1β and lapatinib stimulated proliferation compared with untreated controls, while NRG1β alone was inhibitory. The stimulatory effect of lapatinib plus NRG1β was observable at the first 2 hr time point post drug exposure and was maintained for a full 96-hr time course (Figures 1D and S2A). HGF diminished lapatinib response in HCC1954 cells in a dose-dependent manner, and HGF alone stimulated proliferation (Figures 1E and S2B). Evidence for apoptosis was not observed in SKBR3 cells following lapatinib treatment, but growth curves for HCC1954 show cell death beginning 24 hr after addition of lapatinib, indicating a differential response to HER2 inhibition between these cell types.

Since lapatinib is FDA approved as second-line treatment in combination with capecitabine (Ryan et al., 2008), we performed

(B and C) Isolated plots from (A) of AU565 and HCC1954 cells exposed to NRG1β and HGF, respectively, following lapatinib treatment shows ECM or adhesion proteins influencing ligand-mediated drug resistance. Error bars display SEM, n = 13–15.

(D and E) Mean cell count (n = 2, biological replicates [BR] = 3) derived from live-cell imaging of nuclear-GFP-expressing SKBR3 and HCC1954 cells treated with DMSO, 500 nM lapatinib, 25 ng/mL NRG1β or HGF, and 500 nM lapatinib plus 25 ng/mL NRG1β or HGF over a 96-hr time course. Growth factors were added at time 0, and lapatinib was spiked in at the 24-hr time point. Cell counts normalized to counts at time 0 for each treatment condition.

(F and G) Mean cell count and SEM (n = 3) for AU565 and HCC1954 cells treated for 72 hr with combinations of DMSO, 500 nM lapatinib, 500 μM capecitabine, and 50 ng/mL NRG1β or HGF. Lapatinib significantly decreased cell counts compared with DMSO (****AU565, $p < 0.0001$; ****HCC1954, $p < 0.0001$), and ligand added to lapatinib significantly increased cell count compared with lapatinib alone in both cell lines (***AU565, $p = 0.0009$; ****HCC1954, $p < 0.0001$). Capecitabine significantly decreased the cell count compared with DMSO (**AU565, $p = 0.0091$; ***HCC1954, $p = 0.0002$), but addition of ligand did not significantly alter cell counts. The combination of lapatinib and capecitabine significantly decreased cell counts compared with DMSO (****AU565, $p < 0.0001$; ***HCC1954, $p = 0.0002$), and the addition of ligand significantly increased cell counts compared with the combination alone in both cell lines (**AU565, $p = 0.0044$; **HCC1954, $p = 0.0033$).

ns, not significant.

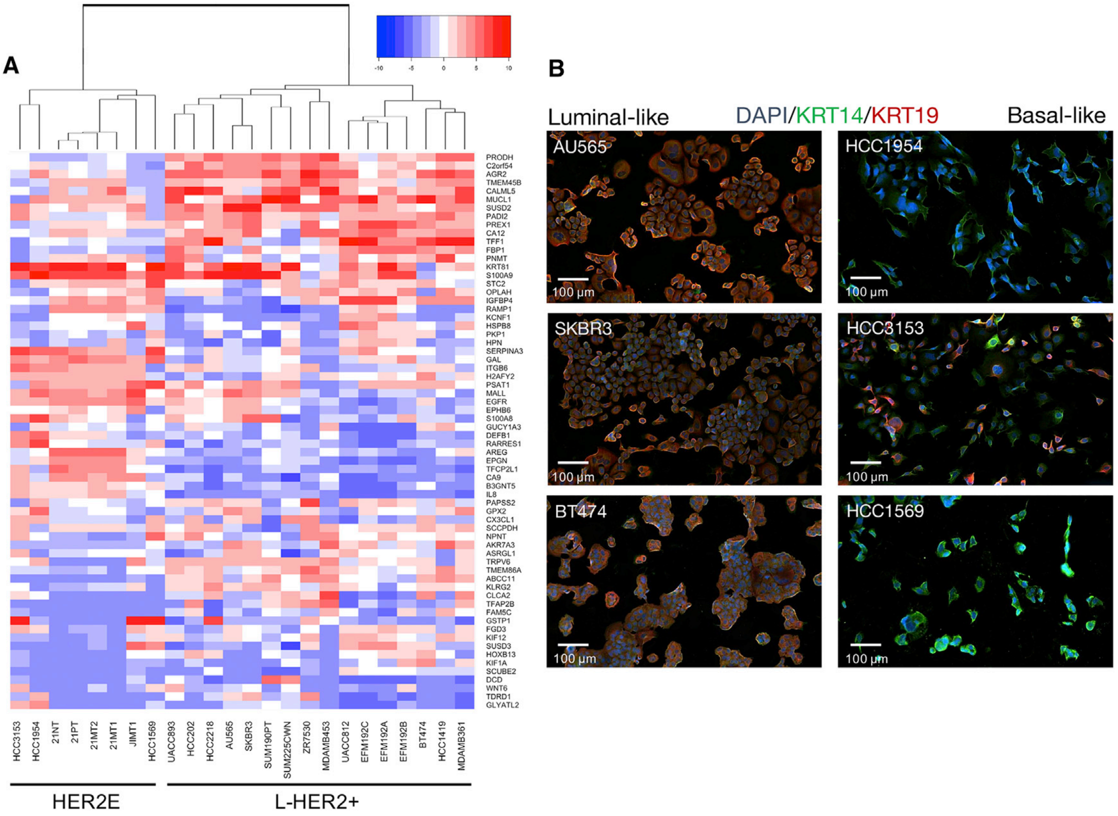

Figure 2. HER2+ Cell Lines Are Sub-classified into Luminal-like L-HER2+ and Basal-like HER2E Phenotypes

(A) mRNA expression clustered heatmap of genes identified by TCGA as significantly different between HER2E and luminal HER2+ patient tumors in a panel of HER2+ breast cancer cell lines. Gene expression was sorted for variance across cell lines; the top 10% (66 genes) are used to cluster the panel.

(B) Representative images of L-HER2+ and HER2E lines immunofluorescently labeled with DAPI (blue), KRT14 (green), and KRT19 (red).

combination treatments with lapatinib, NRG1β, and capecitabine in AU565 or SKBR3 cells, and lapatinib, HGF, and capecitabine in HCC1954 cells to determine the relevance of growth-factor-mediated drug resistance to the clinical use of lapatinib (Figures 1F, 1G, and S3). Lapatinib and capecitabine both decreased cell counts in all cell lines, and the combination of both drugs resulted in a greater reduction of cell count than either agent alone. Addition of NRG1β to the combination of lapatinib and capecitabine significantly reduced the drug combination efficacy in both SKBR3 and AU565. Furthermore, in SKBR3 cells, we observed increased cell counts over untreated controls (Figure S3), consistent with our earlier observation that NRG1β can convert lapatinib treatment into a stimulator of growth. In addition, HGF significantly reduced the effectiveness of the combination of lapatinib and capecitabine in HCC1954 cells (Figure 1G).

HER2+ Subtypes Show Differential Response to NRG1β and HGF

We explored the possibility that differences in the responses of SKBR3 and HCC1954 cells to microenvironmental signals were due to differences in the subtype-specific intrinsic biology of L-HER2+ and HER2E cells. We assessed the subtype specificity of the effects of HGF and NRG1β on drug response in a panel of four cell lines representing the L-HER2+ subtype and four representing the HER2E subtype. We switched to the irreversible HER2 TKI neratinib for these studies since several HER2E cell lines are innately resistant to lapatinib.

We classified cell lines as L-HER2+ or HER2E based on the expression of 302 genes identified by the TCGA as differentially expressed between L-HER2+ and HER2E subtypes (Cancer Genome Atlas Network, 2012). We filtered this gene set for variance across our panel of HER2+ breast cancer cell lines and performed cluster analysis (Figure 2A). The cell line panel clustered into two subtypes with similar expression profiles to patient L-HER2+ and HER2E subtypes, and shared consistent transcriptional similarity with luminal and basal mammary cell types, respectively (Figure 2B). We chose the cell lines JIMT1, HCC-3153, HCC1954, and 21MT1 as models of the HER2E subtype, and EFM192A, BT474, SKBR3, and AU565 as models of the L-HER2+ subtype.

We explored the effects of varying concentrations of neratinib, NRG1β, and HGF across the HER2+ cell line panel. Figure 3A shows that NRG1β attenuated response to neratinib in the L-HER2+ lines but generally not in the HER2E cell lines, particularly at higher concentrations. HGF strongly attenuated response to neratinib in the HER2E lines but had little effect in the L-HER2+ cell lines. This was most evident at the 200 nM dose of neratinib. In many cases, L-HER2+ cells treated for 72 hr with neratinib in

the presence of NRG1β showed a higher average cell count and a higher percentage of proliferating cells than untreated controls (Figures 3A and 3B), consistent with the stimulation of proliferation observed with the combination of NRG1β and lapatinib or lapatinib plus capecitabine.

Subtype Intrinsic Responses to NRG1β and HGF Are Observed in 3D Cultures and Murine Xenografts

We measured the responses of L-HER2+ cell lines (SKBR3, AU565) and HER2E cell lines (HCC1954, 21MT1) grown in 3D Matrigel cultures to determine whether spatial organization and ECM structure altered the effects of HGF and NRG1β. The cells were treated with combinations of NRG1β, HGF, and neratinib (Figure 3C). Cells were generally less responsive to neratinib at baseline in 3D than observed in 2D, as has been reported previously with lapatinib (Weigelt and Bissell, 2008). However, we found that NRG1β and HGF reversed the inhibitory effects of neratinib in L-HER2+ cells and HER2E cells, respectively, in 3D cultures.

We also showed that the subtype-specific effects of microenvironmental signals on HER2-targeted TKIs were present in HER2+ murine xenografts. HER2E JIMT1 and L-HER2+ BT474 breast cancer cells were transplanted subcutaneously into the flank of the hind leg and orthotopically to mammary fat pads. We selected BT474 and JIMT1 cells for this experiment due to their ability to form solid tumors in mice without the need for estrogen pellets (Gu et al., 2016). Induced tumors were implanted with nanodosing microdevices (Jonas et al., 2015) loaded with polyethylene glycol (PEG) control, HER2-TKI, HER2-TKI in combination with HGF and NRG1β, and proteins alone. The implanted tumors were extracted and processed by immunofluorescent histochemistry for analysis of cleaved caspase-3 (CC3) for apoptosis and Ki67 for proliferation after 48 hr of exposure to the various nanodose drug combinations. Lapatinib was used in BT474 and neratinib was used in JIMT1 to adjust for the innate drug sensitivities of each cell line.

Lapatinib decreased proliferation in areas of BT474 xenograft tumors in close proximity to the nanodosing reservoirs, while neratinib mainly increased the rate of apoptosis in JIMT1 tumors (Figures 3D, 3E, and S4). The combination of lapatinib with NRG1β, but not HGF, restored the Ki67 signal to control levels in BT474 tumors (Figure 3D). We were not able to compare apoptosis induced by different treatment conditions in BT474, as the basal levels were extremely low (Figure S4C). In contrast, adding HGF positively affected proliferation and significantly reduced the CC3 levels in JIMT1 tumors (Figures 3E, S4A, and S4B). NRG1β did not significantly increase Ki67 or reduce CC3 levels in the JIMT1 xenografts. These results confirm the cytostatic and cytotoxic impact of HER2 inhibition that we observed *in vitro* in SKBR3 and HCC1954 cells, respectively (Figures 1D and 1E).

HER2+ Subtypes Differ in Signaling Biology

We analyzed transcriptional profiles using RNA sequencing (RNA-seq) (Daemen et al., 2013) and protein profiles using reverse-phase protein arrays (RPPA) (Korkola et al., 2015) for HER2E and L-HER2+ cells to identify molecular processes that might account for the observed differences in response to microenvironmental signals. We performed gene set enrichment analysis (GSEA) on RNA-seq profiles measured for eight L-HER2+ and eight HER2E lines at baseline culture conditions (Table 1). An unbiased query of the Hallmarks library of gene signatures showed that the "KRAS Signaling Up" gene set was significantly enhanced in HER2E cells compared with L-HER2+ cells (Figure S5A). In contrast, one of the most significant gene sets upregulated in the reverse comparison of L-HER2+ versus HER2E was the "KRAS Signaling Down" gene set, indicating that this pathway is differentially regulated between the subtypes. We also observed that *FOXA1*, the inducible transcription factor that binds to the promoter region of HER3 (Ni et al., 2011), was markedly higher in L-HER2+ expression compared with HER2E, as was *ERBB3* expression. Conversely, we found that *EGFR* and *MET* expression was significantly higher in HER2E than in L-HER2+ (Figures 4A, 4B, and 4D). Figure 4A plots *ERBB3* against *MET* expression to highlight the difference in expression of the NRG1β and HGF receptors between L-HER2+ and HER2E for the entire HER2+ cell line panel. The same relative expression trends for *MET* and *ERBB3* were present in gene expression profiles for L-HER2+ and HER2E human tumors analyzed by TCGA (Figure 4C).

Western analyses showed that treatment of both HER2 subtypes with lapatinib reduced levels of pHER3 and pAKT, and that pAKT expression in both subtypes could be restored by adding NRG1β (Figure S5B). However, NRG1β restored pS6 levels, an indicator of active mitogenic signaling (Thomas et al., 1979), only in the L-HER2+ lines, while HGF restored pS6 levels only in HER2E lines. Taken together, these analyses suggest that HER2E lines preferentially rely on MAPK signaling and that L-HER2+ lines preferentially rely on PI3K signaling, and that these pathways separately converge on S6K to execute effects on proliferation.

Our data and previous reports (Donnelly et al., 2014) suggest that HER2E lines preferentially rely on MET and MAPK signaling and that L-HER2+ lines preferentially rely on HER3 and phosphatidylinositol 3-kinase (PI3K) signaling. We tested this possibility in siRNA knockdown experiments and found that HER2E cells do not depend as strongly on HER3 as do L-HER2+ lines (Figure S6A). We also reanalyzed previously published RPPA measurements of the temporal responses of L-HER2+ and HER2E cell lines to treatment with 250 nM lapatinib (Korkola et al., 2015) and found that lapatinib preferentially inhibited activity of PI3K-mTORC pathway constituents in AU565 (L-HER2+) compared with HCC1954 (HER2E), and inhibited activity of EGFR and MEK in HCC1954 compared with AU565 (Figure 4E). We further assessed reliance on PI3K and MAPK pathways in L-HER2+ and HER2E lines by measuring the responses of the HER2E cell lines JIMT1, HCC1954, 21MT1, 21PT1, and HCC3153, and the L-HER2+ lines SKBR3, BT474, AU565, MDAMB361, and EFM192A to nine different concentrations of lapatinib, the MEK inhibitor trametinib, and the combination. Figure 4F shows that the L-HER2+ lines were less sensitive to the MEK inhibitor and more sensitive to lapatinib than HER2E lines. HER2E lines were more sensitive to trametinib than L-HER2+ lines, and the combination of lapatinib and trametinib resulted in significantly decreased cell viability in comparison with each agent alone in HER2E cells.

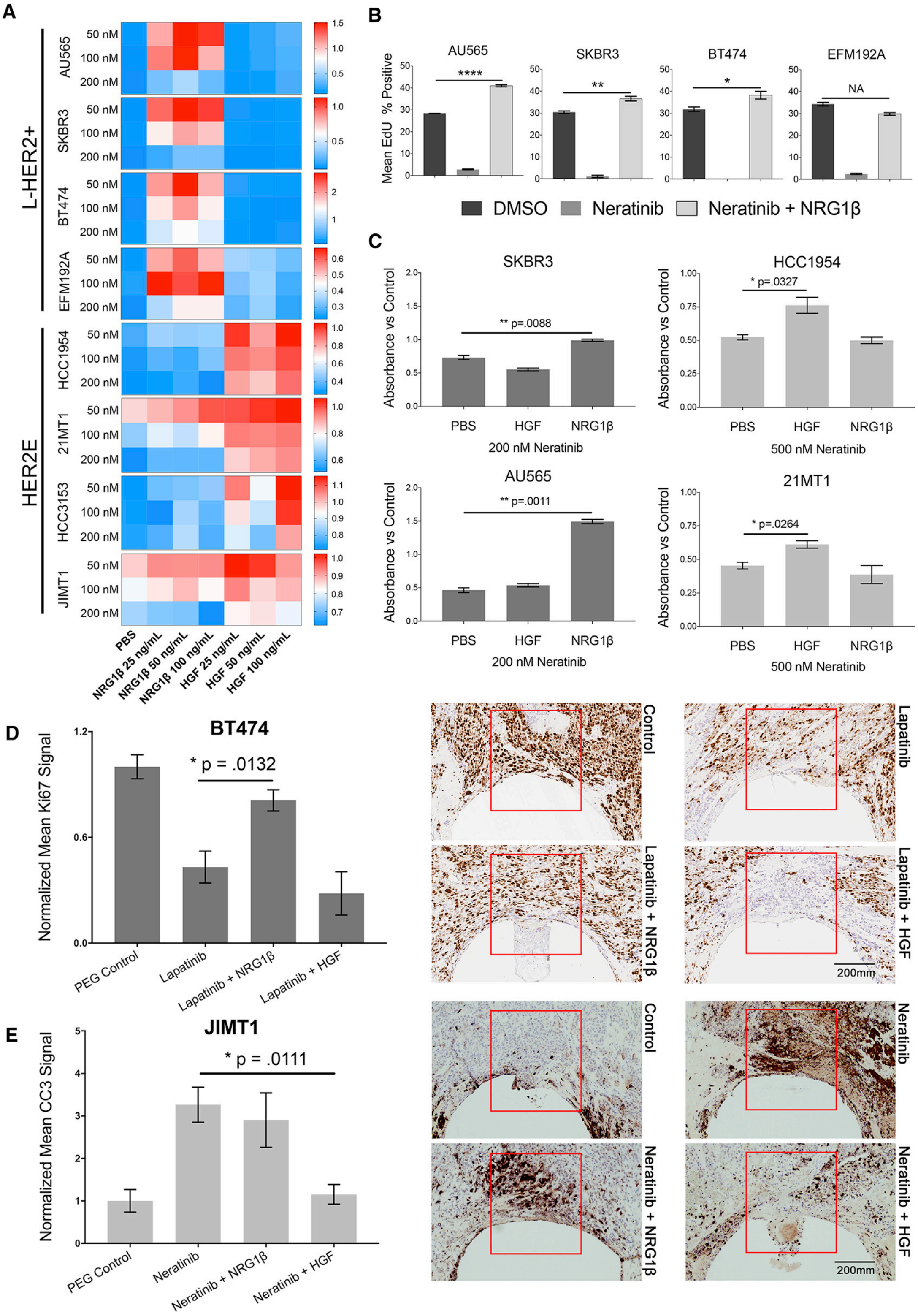

Figure 3. HER2+ Breast Cancer Cell Lines Exhibit a Subtype Intrinsic Proliferative Response to NRG1β and HGF under Lapatinib or Neratinib Treatment

(A) Heatmap of mean cell count (n = 3) of 8 HER2+ cell lines exposed to a dose range of neratinib and three concentrations of NRG1β and HGF. Each value is normalized to the mean cell count of the corresponding DMSO-treated control. Scale to the right indicates the relative cell count ratio between drug-treated and untreated controls.

(legend continued on next page)

Table 1. L-HER2+ and HER2E Cell Lines Exhibit Differential Regulation of MAPK and Estrogen Receptor Pathways

Enriched Gene Sets	L-HER2 or HER2E	Set Size	ES	NES	Nominal P Value	FDR Q Value	FWER Q Value	Rank at MAX
HALLMARK_ESTROGEN_RESPONSE_LATE	L-HER2	197	0.399	1.435	0.003	0.080	0.179	3,898
HALLMARK_KRAS_SIGNALING_DN	L-HER2	196	0.376	1.340	0.003	0.098	0.75	3,186
CHARAFE_BREAST_CANCER_LUMINAL_VS_ MESENCHYMAL_UP	L-HER2	409	0.718	2.740	0.000	0.000	0	4,656
CHARAFE_BREAST_CANCER_LUMINAL_VS_BASAL_UP	L-HER2	331	0.723	2.728	0.000	0.000	0	3,968
DOANE_BREAST_CANCER_ESR1_UP	L-HER2	105	0.734	2.410	0.000	0.000	0	3,184
SMID_BREAST_CANCER_ERBB2_UP	L-HER2	137	0.640	2.171	0.000	0.000	0	3,999
LIM_MAMMARY_LUMINAL_MATURE_UP	L-HER2	112	0.585	1.948	0.000	0.004	0.108	4,358
SMID_BREAST_CANCER_LUMINAL_B_UP	L-HER2	160	0.529	1.846	0.000	0.019	0.512	5,542
HALLMARK_EPITHELIAL_MESENCHYMAL_TRANSITION	HER2E	196	−0.674	−2.265	0.000	0.000	0	4,041
HALLMARK_TNFA_SIGNALING_VIA_NFKB	HER2E	189	−0.671	−2.226	0.000	0.000	0	4,687
HALLMARK_KRAS_SIGNALING_UP	HER2E	195	−0.579	−1.932	0.000	0.000	0	4,596
CHARAFE_BREAST_CANCER_LUMINAL_VS_BASAL_DN	HER2E	421	−0.804	−2.875	0.000	0.000	0	3,540
CHARAFE_BREAST_CANCER_LUMINAL_VS_ MESENCHYMAL_DN	HER2E	431	−0.793	−2.855	0.000	0.000	0	3,662
WU_CELL_MIGRATION	HER2E	175	−0.675	−2.230	0.000	0.000	0	3,196
VANTVEER_BREAST_CANCER_ESR1_DN	HER2E	226	−0.651	−2.191	0.000	0.000	0	5,062
GO_EXTRACELLULAR_STRUCTURE_ORGANIZATION	HER2E	297	−0.553	−1.934	0.000	0.040	0.062	3,607

Table of selected significantly enriched gene sets (false discovery rate [FDR] q value <25% and nominal p value <0.01) for GSEA comparisons of eight L-HER2+ (AU565, SKBR3, BT474, EFM192A, EFM192B, EFM192C, UACC812, ZR7530) and eight HER2 cell lines (JIMT1, 21MT1, 21MT2, 21PT, 21NT, HCC1954, HCC3153, HCC1569). Table shows gene sets enriched in comparison of L-HER2+ versus HER2E and in the reverse comparison. ES, enrichment score; NES, normalized enrichment score; FWER, familywise error rate.

Countering Microenvironment-Mediated Resistance

Our studies suggest that the HGF-mediated attenuation of response of HER2E cells to lapatinib or neratinib is due to the constitutive high level of expression of MET in HER2E cells, which allows HER2E cells to utilize HGF to escape lapatinib or neratinib inhibition. This raised the possibility that combined treatment of HER2E cells with neratinib and the MET targeting TKI crizotinib could block signaling through MET to abrogate resistance to neratinib. Figure 5A demonstrates that this is the case; crizotinib eliminated HGF-mediated neratinib resistance in four HER2E lines but did not block NRG1β-mediated lapatinib resistance in four L-HER2+ lines.

The mechanism by which NRG1β combined with lapatinib or neratinib results in growth stimulation in L-HER2+ cells appears to be a multistep process that begins with the previously reported translocation of HER3 from the endocytic compartment of the cytoplasm to the cell surface, which is triggered by a drug-induced reduction in pAKT levels (Amin et al., 2010; Sergina et al., 2007). Exogenous NRG1β binding to HER3 then stabilizes increased numbers of HER2-HER3 heterodimers at the cell surface. HER2E cell lines do not depend on PI3K signaling (Figure 4E), so the PI3K-inhibition-induced translocation of HER3 to the cell surface does not occur. We confirmed this using proximity ligation assays (PLA) to assess lapatinib-induced differences in HER2-HER3 dimerization on the cell surface of the L-HER2+ line SKBR3, and the HER2E line HCC1954 treated with combinations of lapatinib and NRG1β. Figure 5B shows a significant increase in heterodimers on the cell surface in L-HER2+ lines under exposure to the combination of lapatinib and NRG1β following 48 hr of treatment. No such increase was observed in HCC1954 (Figure 5C). Thus, L-HER2+ cells express high levels of NRG1β-HER2-HER3 complexes after lapatinib treatment while HER2E cells do not.

This observation does not explain the increased proliferation observed immediately after TKI treatment in L-HER2+ cells, since HER2 kinase activity should still be blocked. However, structural studies of HER-family kinase domains (Wood et al., 2004; Aertgeerts et al., 2011) suggest that the activation of HER2-HER3 heterodimers by NRG1β changes the conformation of the ATP-binding pocket of HER2 targeted by TKI so that their binding is reduced (Novotny et al., 2016). We reasoned that treatment of the NRG1β-HER2-HER3 complex with pertuzumab

(B) Mean percentage of EdU-positive cells and SEM (n = 3) in four L-HER2+ cell lines treated in (A) with DMSO, 100 nM neratinib, and 100 nM neratinib plus 25 ng/mL NRG1β. Neratinib plus NRG1β treatment results in significantly increased EdU positivity compared with DMSO treatment in 3 out of 4 cell lines (****AU565, $p < 0.0001$; **SKBR3, $p = 0.0068$; *BT474, $p = 0.0397$).

(C) Mean absorbance measurements and SEM (n = 2, BR = 3) of alamar blue stains from four cell lines in 3D Matrigel assays following 96 hr treatments of 200 or 500 nM neratinib, with and without 50 ng/mL NRG1β and HGF. Absorbance values are shown as the ratio of drug-treated to untreated control.

(D and E) Representative images and quantification of xenograft tumor response to local delivery of drugs alone and in combination with NRG1β and HGF proteins. Reservoirs loaded with pure PEG polymer served as a control. Sectioned tissue surrounding the implantable nanodosing device (red boxes) is stained for CC3 and Ki67 to assess apoptosis and proliferation, respectively. Graphs show mean and SEM normalized signal intensity of the tumor region adjacent to treatment reservoirs (BT474, n = 4; JIMT1, n = 3).

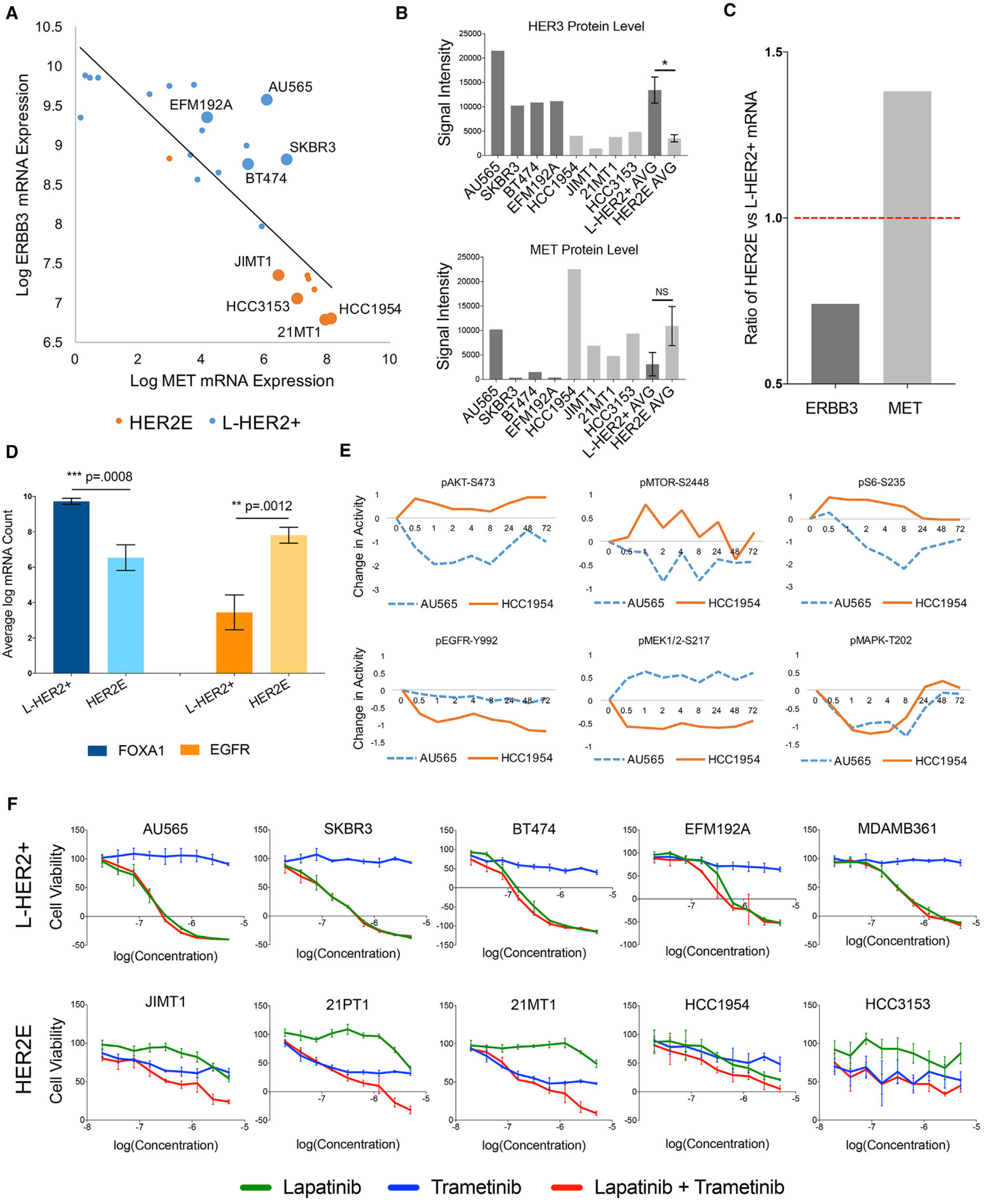

Figure 4. HER2E and L-HER2+ Lines Show Differential Reliance on MAPK and PI3K Signaling

(A) Log-scale mRNA expression of *ERBB3* and *MET* in a panel of HER2+ cell lines. Enlarged spots indicate cell lines used in Figure 3A.

(B) Quantification of western blot protein analysis of HER3 and MET levels in eight cell lines (BR = 3). Average HER3 levels are significantly reduced in HER2E compared with L-HER2+ (*p = 0.012).

(legend continued on next page)

might disrupt the dimers and restore the conformation of the ATP-binding pocket and increase TKI-binding efficiency. Figure 5A shows that this is the case, since pertuzumab significantly reduced NRG1β-mediated lapatinib resistance in four L-HER2+ cell lines but failed to reduce HGF-mediated neratinib resistance in four HER2E lines. Pertuzumab binds to the extracellular domain II of HER2 and is reported to function by inhibiting HER2-activating binding partners such as EGFR and HER3 via steric hindrance (Franklin et al., 2004).

We also tested the possibility that trastuzumab binding to domain IV of HER2 (Cho et al., 2003) would perform the same function by testing the efficacy of trastuzumab treatment in combination with lapatinib and NRG1β in L-HER2+ cells. This is important since neratinib and lapatinib are now being tested clinically in combination with trastuzumab (de Azambuja et al., 2014). In contrast to pertuzumab, trastuzumab did not abrogate NRG1β-mediated resistance and caused a small decrease in cell count as a single agent (Figure S7).

Pertuzumab and Lapatinib Combinations in L-HER2+ Cells Treated with NRG1β

We performed mass spectrometry proteomics on HER3 immunoprecipitates to investigate why pertuzumab inhibited the resistance effect of NRG1β but showed no effect as a monotherapy in L-HER2+ cell lines. We performed bead-based HER3 immunoprecipitation on cell lysates from L-HER2+ AU565 cells treated with combinations of lapatinib, NRG1β, and pertuzumab for 48 hr and quantified proteins that co-precipitated with HER3 (Figure 6A). We filtered the data for proteins that bound to HER3 following phosphorylation of C-terminal regions by HER2, such as subunits of PI3K. The presence or absence of these HER3-binding proteins served as markers of signaling activity of HER2-HER3 dimers. We found that lapatinib treatment significantly reduced the amount of protein bound to HER3 compared with untreated controls. Addition of NRG1β to lapatinib restored the levels of proteins bound to HER3 to control levels. Further addition of pertuzumab to NRG1β and lapatinib eliminated the effect of NRG1β, and resulted in decreased levels of protein bound to HER3 compared with untreated controls. These data demonstrate that pertuzumab restores sensitivity to lapatinib inhibition by blocking phosphorylation of HER3 and reducing interaction with other proteins that bind to HER3. However, pertuzumab did not decrease binding of HER2, which co-precipitated with HER3 in 20-fold greater quantity when treated with pertuzumab.

DISCUSSION

Our goal in this study was to identify microenvironmental factors that drive resistance to the HER2-targeted TKIs in HER2+ breast cancers. We used MEMA technology to identify the specific soluble and matrix factors from the microenvironment that alter the TKI responses in HER2+ breast cancer cells. The power of the platform is its ability to efficiently assess the effects of thousands of different combinatorial microenvironments in multiple cell lines. The platform is generally applicable to assessment of the impact of the microenvironment on any phenotype that can be revealed using fluorescent reporters and quantitative imaging.

Our studies showed that the L-HER2+ and HER2E breast cancer subtypes defined by TCGA differ fundamentally in how they engage the microenvironment. These differences derive from preferential dependence of L-HER2+ cells on HER3 expression and PI3K signaling and preferential dependence of HER2E cells on MET expression and MAPK signaling. These HER2+ subtype differences manifest, even in the absence of microenvironmental signals, as differences in biological and molecular responses to HER2 and MAPK targeted inhibitors as illustrated in Figures 4E and 4F. However, they also lead to HER2+ subtype-specific differences in the microenvironmental signals that alter response to TKIs.

Understanding and managing the interaction of HER2E cells with the microenvironment is straightforward. These cells express high levels of MET and depend on downstream MAPK signaling. High levels of HGF activate MET signaling through MAPK, thereby reducing sensitivity to lapatinib or neratinib. We show that the inhibitory effects of HGF on TKI response can be blocked with crizotinib. This had been independently reported by Settleman et al. in the HER2E cell line, HCC1954 (Wilson et al., 2012); however, we show that this is a general property of the HER2E subtype not limited to a single cell line.

The interaction of L-HER2+ cells with microenvironmental signals is more complicated. This is illustrated by our observation that L-HER2+ cells treated with lapatinib or neratinib proliferated more than untreated control cells when NRG1β was present (Figures 1D, S2, 3A, and 3B). In other words, NRG1β converted lapatinib or neratinib into stimulatory drugs. This proliferative stimulation was even sufficient to overcome the inhibitory effects of the chemotherapeutic drug capecitabine in an *in vitro* setting (Figure 1F). We believe that this enhancement of proliferation involves multiple events. The process begins with TKI-induced inhibition of PI3K signaling that stimulates translocation of cytosolic HER3 to the cell surface (Sergina et al., 2007) where it forms heterodimers with HER2 or EGFR (Arteaga et al., 2011). High levels of NRG1β stabilize HER-HER3 heterodimers on the cell surface and cause a conformation change in the HER2 kinase domain that diminishes lapatinib or neratinib binding (Novotny et al., 2016). The end result is an increase in the number of HER2-HER3 heterodimers that are unchecked by the TKI so that the cells are actually stimulated to proliferate more rapidly than cells that receive no drug treatment. HER2E cells appear to lack the HER3 feedback mechanism so NRG1β does not

(C) Ratio of *ERBB3* and *MET* mRNA expression in human breast cancer tumors defined as HER2E and L-HER2+ by TCGA.
(D) Mean log mRNA counts and SEM (n = 8) of eight L-HER2+ cell lines and eight HER2 lines and expression of FOXA1 and EGFR.
(E) RPPA time course of AU565 and HCC1954 cells treated with 250 nM lapatinib. The y axis shows mean (n = 3) signal intensity of each phospho-protein versus its respective total protein, with each signal normalized to its DMSO-treated control cohort at each time point, representing change in protein activity over 72 hr of treatment. The top three proteins are canonical constituents of the PI3K/MTOR pathway, the bottom three are canonical constituents of the MAPK pathway.
(F) GI 50 graphs and SEM (n = 3) of CTG assays from HER2E and L-HER2+ cell lines treated for 72 hr with a dose range of lapatinib, trametinib, and the combination.

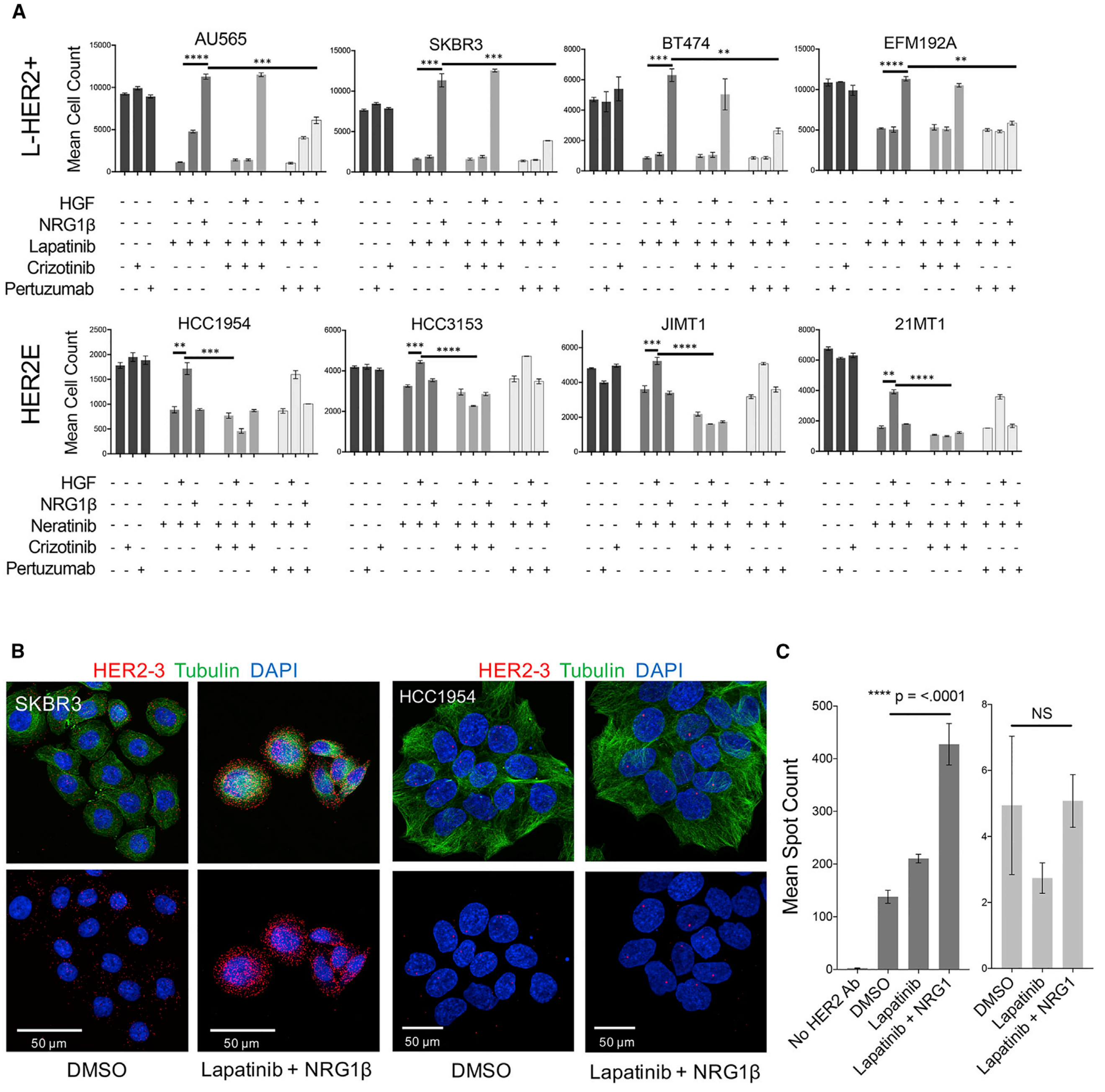

Figure 5. L-HER2+ and HER2E Lines Differ in Their Resistance Mechanisms to HER2 Inhibition

(A) Mean cell count and SEM (n = 3) of eight HER2+ cell lines treated for 72 hr with combinations of 500 nM lapatinib, 500 nM crizotinib, 30 μg/mL pertuzumab, 50 ng/mL NRG1β, 50, and ng/mL HGF. PBS, DMSO, and human IgG isotype control were used as controls for growth factors, TKIs, and pertuzumab, respectively. Addition of NRG1β to lapatinib results in significantly increased cell counts in each L-HER2+ line compared with drug alone (****AU565, $p < 0.0001$; ***SKBR3, $p = 0.0003$; ***BT474, $p = 0.0002$; ****EFM192A, $p < 0.0001$). Addition of HGF to neratinib results in significantly increased cell count in each HER2E line compared with drug alone (**HCC1954, $p = 0.0037$; ***HCC-3153, $p = 0.0002$; ***JIMT1, $p = 0.0001$; **21-MT1, $p = 0.0045$). Addition of pertuzumab to lapatinib plus NRG1β results in significantly decreased cell counts in each L-HER2+ line compared with lapatinib plus NRG1β alone (***AU565, $p = 0.0005$; ***SKBR3, $p = 0.0008$; **BT474, $p = 0.0013$; **EFM192A, $p = 0.0001$). Addition of crizotinib to neratinib plus HGF results in significantly decreased cell counts in each HER2E line compared with neratinib plus HGF alone (***HCC1954, $p = 0.0006$; ****HCC-3153, $p < 0.0001$; ****JIMT1, $p < 0.0001$; ****21-MT1, $p < 0.0001$).

(B) Maximum projection fluorescent images of SKBR3 and HCC1954 cells treated for 48 hr with combinations of 500 nM lapatinib and 12.5 ng/mL NRG1β. Cell nuclei imaged with DAPI (blue), β-tubulin (green), and HER2-HER3 heterodimers (red) imaged by PLA.

(C) Mean and SEM of PLA spot counts for SKBR3 (n = 79, 93, 150, 54) and HCC1954 (n = 52, 60, 54) treated for 48 hr with DSMO, 12.5 ng/mL NRG1β, 500 nM lapatinib, and the combination. p value shows unpaired t test of significance. NS, not significant.

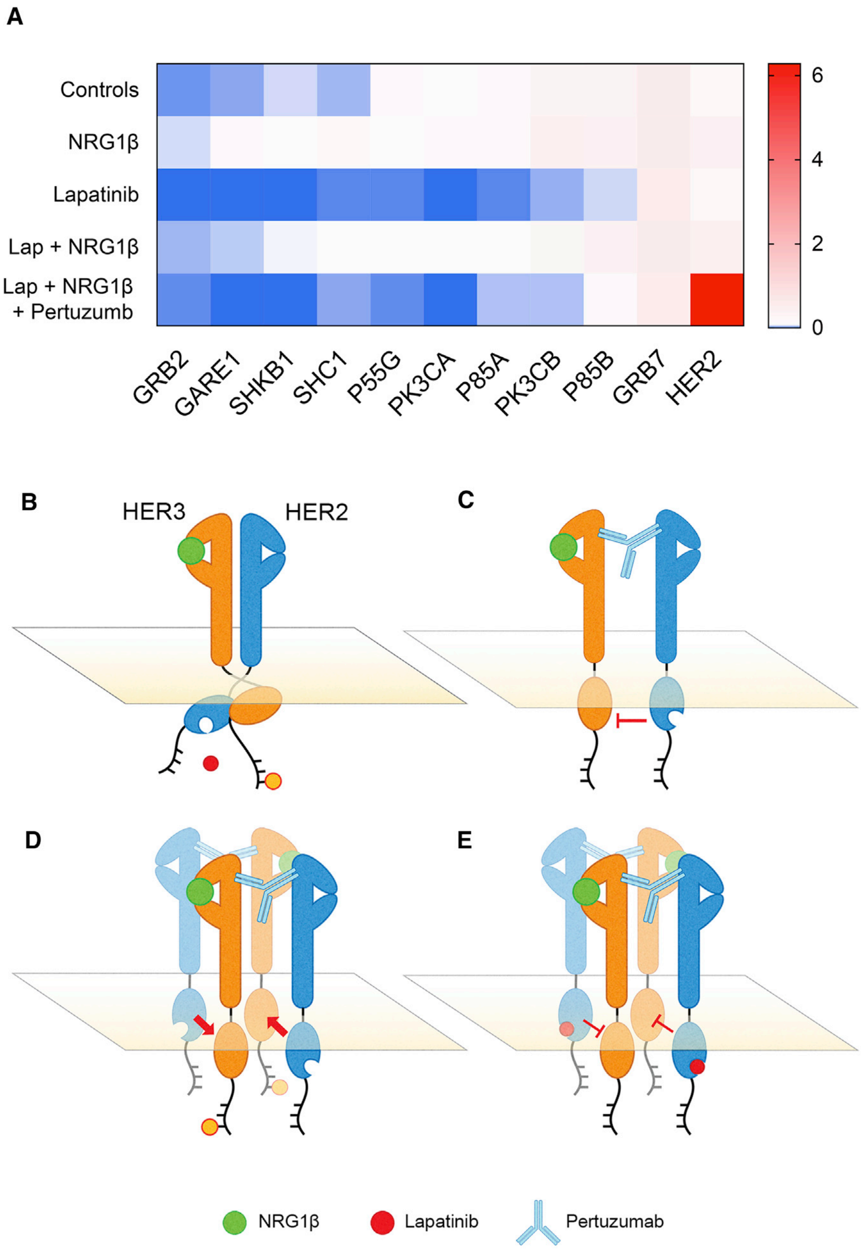

Figure 6. Association of PI3K Subunits with HER3 Regulated by NRG1 and HER2 Targeting Drugs

(A) Heatmap of detection counts from mass spectrometry proteomic analysis of HER3 immunoprecipitation from AU565 cell lysate following 48 hr of treatment with 500 nM lapatinib, 50 ng/mL NRG1β, 25 μg/mL pertuzumab, and selected combinations. Each count is normalized to the detected quantity of HER3 in each treatment sample. Scale shows the ratio of individual protein count to HER3 count (BR = 3).

(B) A simplified model of HER2 and HER3 dimerization on the cell surface when activated by NRG1β. This structure alters the conformation of the ATP-binding pocket on the HER2 cytoplasmic kinase domain to one to which lapatinib cannot readily bind.

(C) Pertuzumab inhibits *cis*-phosphorylation of HER3 via separation of the cytoplasmic kinase domains. This structure returns the HER2 kinase domain conformation to one to which lapatinib can bind. Note that receptors remain linked under pertuzumab treatment.

(D) Higher-order receptor structures overcome inhibition by pertuzumab via *trans*-phosphorylation of HER3 (orange dot).

(E) The proposed mechanism for how pertuzumab and lapatinib combine to overcome *cis*- and *trans*-phosphorylation of HER3, respectively.

restore proliferation under lapatinib or neratinib treatment in these cells.

We confirmed previous reports that NRG1β-mediated resistance in HER2+ cells could be reversed by adding pertuzumab (Leung et al., 2015). Our studies showed that this beneficial effect is confined to the L-HER2+ subtype. Our data show that pertuzumab but not trastuzumab alters the HER2-HER3 conformation to restore TKI binding to the HER2 kinase domain. We also found that pertuzumab had no efficacy as a single agent, despite its reported ability to interfere with HER2 dimerization (Harbeck et al., 2013). Instead, we found that pertuzumab increased proliferation when combined with NRG1β. Mass spectrometry analysis showed that pertuzumab reduced the association of HER3 with PI3K pathway mediators in cells treated with lapatinib and NRG1β, but increased the total amount of HER2 associated with HER3 in the presence of lapatinib and NRG1β. Structural studies of HER2-HER3 dimers by Zhang et al. (2012) suggest that this is because HER2 has the capacity to trans-phosphorylate HER3 of neighboring heterodimers when both HER2 and HER3 are highly overexpressed, which occurs as a result of HER2 amplification and TKI-mediated translocation of HER3 to the cell surface. Figure 6B summarizes the *trans*- and *cis*-phosphorylation mechanisms by which NRG1β interacts with HER2-HER3 heterodimers to activate mitogenic signaling. This model suggests that complete inhibition of the HER2-HER3 signaling in L-HER2+ cells in high NRG1β environments requires the combination of TKI and pertuzumab to inhibit HER2 kinase activity, block the HER2 conformation change, and overcome both *cis*- and *trans*-activation of HER3. Other strategies that might be deployed to defeat NRG1β-mediated resistance include co-treatment with drugs to inhibit AKT-mediated upregulation of HER3, treatment with antibodies targeting NRG1β (Hegde et al., 2013), or designing small-molecule inhibitors that are effective against the HER2 kinase in the altered configuration (Novotny et al., 2016).

Our studies raise the possibility that responses to TKIs may vary between different anatomical metastatic sites since the levels of HGF and/or NRG1β expression differ between sites to

which HER2+ cancers may metastasize. Both HGF and/or NRG1β are highly expressed by cancer associated fibroblasts in the breast (Capparelli et al., 2015; Tyan et al., 2011) and high expression has been associated with poor prognosis and drug resistance (Veenstra et al., 2016; Straussman et al., 2012; Lin et al., 2014). HGF also is highly expressed in the liver and lung (Uhlén et al., 2015), common sites of HER2E metastasis, while NRG1β is highly expressed at common sites of L-HER2+ metastasis, including the lung, lymph node, and brain (Uhlén et al., 2015; Law et al., 2004). The concentrations of NRG1β and HGF that we found to decrease TKI efficacy (12.5–200 ng/mL) are similar to that found in human tissue. The concentration of HGF secreted by patient-derived bone marrow stem cells over 48 hr was reported to be 2–12 ng/mL (Takai et al., 1997) and the concentration of HGF was reported to be 0.5–11 ng/mL in pleural effusions from cancer patients (Eagles et al., 1996). The concentration of NRG1β was reported to be 5–700 ng/mL in serum (Moondra et al., 2009) and 50–10,000 ng/mL in pulmonary fluid from patients with acute lung injury (Finigan et al., 2011). Jain and co-workers recently reported that NRG1β expression in the brain microenvironment was associated with resistance to PI3K inhibitors in L-HER2+ brain metastases, providing further support that metastatic site-specific microenvironments can drive TKI resistance in specific cancer cell types (Kodack et al., 2017).

Our findings raise the possibility that clinical control of HER2+ breast tumors with HER2-targeted TKIs lapatinib and neratininb may be improved by HER2+ subtype-specific strategies to counter resistive microenvironmental signals. Specifically, our data suggest TKI plus pertuzumab for L-HER + breast cancers and TKI plus crizotinib for HER2E breast cancers could be effective treatment strategies. Such strategies would likely apply to the approved TKIs neratinib and lapatinib, as well as to the newer HER2-targeted TKIs under development. Clinical impact may be observed in advanced cancers where the tumor cells encounter distant microenvironments that manifest high levels of HGF or NRG1β. Careful attention to microenvironmental factors in clinical studies with neratinib and lapatinib in combination with chemotherapy or trastuzumab is warranted, since our findings suggest that HGF- and NRG1β-mediated resistance is still operational in these settings. Furthermore, our results suggest caution in the implementation and interpretation of results from basket trials in which all patients with a specified genomic aberration are treated with the same therapy independently of tumor type or subtype. Our data suggest that the epigenetic status can significantly modify oncogene function (HER2 in our case) and therapeutic response.

STAR★METHODS

Detailed methods are provided in the online version of this paper and include the following:

- KEY RESOURCES TABLE
- CONTACT FOR REAGENT AND RESOURCE SHARING
- EXPERIMENTAL MODELS AND SUBJECT DETAILS
  - Breast Cancer Cell Lines
  - Murine Models
- METHOD DETAILS
  - Experimental Design
  - Drug Treatment
  - Fluorescence Cell Line Generation
  - MicroEnvironment MicroArrays
  - Immunofluorescent Histochemistry and Fluorescent Imaging
  - EdU Incorporation
  - Live Cell Imaging
  - 3D Cultures
  - Proximity Ligation Assays
  - Liposomal siRNA Transfection
  - Protein Expression by RPPA or Immunoblots
  - Immunoprecipitation Mass Spectrometry
  - RNAseq
  - Murine Model Experiments
- QUANTIFICATION AND STATISTICAL ANALYSIS
  - RNAseq Hierarchal Clustering
  - Gene Set Enrichment Analysis
- DATA AND SOFTWARE AVAILABILITY
  - MicroEnvironment MicroArray Data
  - Immunoblot Supplement

SUPPLEMENTAL INFORMATION

Supplemental Information includes seven figures and one data file and can be found with this article online at https://doi.org/10.1016/j.cels.2018.02.001.

ACKNOWLEDGMENTS

Research reported in this publication was supported by the NIH Common Fund Library of Network Cellular Signatures (LINCS) grant HG008100 (J.W.G., G.B.M., L.M.H., and J.E.K.) and the Susan G. Komen Foundation award SAC110012 (J.W.G.). S.S.W. was supported by the National Cancer Institute of the NIH under award number F31CA200322. Additional support was provided by the Knight Cancer Institute NCI grant 5P30CA069533-16 (J.W.G.), NIH grant CA195469 (J.W.G), and the Prospect Creek Foundation (J.W.G. and J.E.K.). O.J. was supported by the Brigham Research Institute, Director's Transformative Award. The content is solely the responsibility of the authors and does not necessarily represent the official views of the National Institutes of Health nor does it reflect the position or the policy of the Government, and no official endorsement should be inferred. Mass spectrometric analysis was performed by the OHSU Proteomics Shared Resource with partial support from NIH core grants P30EY010572 and P30CA069533, and shared instrument grant S10OD012246. Microscopy and image cytometry was supported by the Knight Cancer Institute Multiscale Microscopy Core with partial support from the OHSU Center for Spatial Systems Biomedicine (OCSSB), and the MJ Murdock Charitable Trust. RPPA data were generated at the RPPA Core facility at the MD Anderson Cancer Center supported by NCI grant CA16672. We would like to thank Dr. Mark LaBarge (malabarge@lbl.gov) at Lawrence Berkeley National Labs (now at City of Hope) for his contributions to the development of the MEMA platform. In addition, we would like to thank Johann Gagnon-Bartsch for his work on array data normalization and the RUV pipeline. We would also like to thank Dr. Wassana Yantasee, Shenda Gu, and Taylor Ishida for their generous assistance with the murine xenograft project, and Ting Zheng for her assistance with proximity ligation assays.

AUTHOR CONTRIBUTIONS

The manuscript was written by S.S.W., J.W.G., and J.E.K.; J.W.G., S.S.W., and J.E.K. conceived of the project and experimental approach; J.W.G. and J.E.K. supervised the project and provided material support. Image analysis of MEMA and PLA assays was provided by M.N. Advanced data analysis of the MEMA assay and RNA-seq was provided by M.D. and L.M.H. E.B.

developed and implemented tools for accurate tracking of all metadata for the MEMA experiments. S.S.W. performed MEMA and imaging with support from T.L., W.T., R.S., and D.K. M.L. performed 3D Matrigel experiments. K.C. provided the PLA assay and support. D.S. provided OMERO integration and imaging support. O.J., M.W., and Z.T. performed and analyzed murine xenograft nanodosing experiments. G.B.M. provided RPPA experimental data and analysis. O.J. and M.W. performed mouse studies and analysis. J.E.K. performed drug combination screens. All other experiments were the work of S.S.W.

DECLARATION OF INTERESTS

J.W.G. serves on advisory boards to KromaTid, New Leaf Ventures, PDX Pharmaceuticals, and Convergent Genomics. He has licensed technologies to Abbott Diagnostics and Cepheid. J.E.K. is a cofounder and advisor to Convergent Genomics.

Received: February 21, 2017
Revised: August 16, 2017
Accepted: February 2, 2018
Published: March 14, 2018

REFERENCES

Acerbi, I., Cassereau, L., Dean, I., Shi, Q., Au, A., Park, C., Chen, Y.Y., Liphardt, J., Hwang, E.S., and Weaver, V.M. (2015). Human breast cancer invasion and aggression correlates with ECM stiffening and immune cell infiltration. Integr. Biol. (Camb.) *7*, 1120–1134.

Aertgeerts, K., Skene, R., Yano, J., Sang, B.C., Zou, H., Snell, G., Jennings, A., Iwamoto, K., Habuka, N., Hirokawa, A., et al. (2011). Structural analysis of the mechanism of inhibition and allosteric activation of the kinase domain of HER2 protein. J. Biol. Chem. *286*, 18756–18765.

Allan, C., Burel, J.M., Moore, J., Blackburn, C., Linkert, M., Loynton, S., Macdonald, D., Moore, W.J., Neves, C., Patterson, A., et al. (2012). OMERO: flexible, model-driven data management for experimental biology. Nat. Methods *9*, 245–253.

Amin, D.N., Sergina, N., Ahuja, D., McMahon, M., Blair, J.A., Wang, D., Hann, B., Koch, K.M., Shokat, K.M., and Moasser, M.M. (2010). Resiliency and vulnerability in the HER2-HER3 tumorigenic driver. Sci. Transl. Med. *2*, 16ra17.

Arteaga, C.L., Sliwkowski, M.X., Osborne, C.K., Perez, E.A., Puglisi, F., and Gianni, L. (2011). Treatment of HER2-positive breast cancer: current status and future perspectives. Nat. Rev. Clin. Oncol. *9*, 16–32.

de Azambuja, E., Holmes, A.P., Piccart-Gebhart, M., Holmes, E., Di Cosimo, S., Swaby, R.F., Untch, M., Jackisch, C., Lang, I., Smith, I., et al. (2014). Lapatinib with trastuzumab for HER2-positive early breast cancer (NeoALTTO): survival outcomes of a randomised, open-label, multicentre, phase 3 trial and their association with pathological complete response. Lancet Oncol. *15*, 1137–1146.

Cancer Genome Atlas Network. (2012). Comprehensive molecular portraits of human breast tumours. Nature *490*, 61–70.

Capparelli, C., Rosenbaum, S., Berger, A.C., and Aplin, A.E. (2015). Fibroblast-derived neuregulin 1 promotes compensatory ErbB3 receptor signaling in mutant BRAF melanoma. J. Biol. Chem. *290*, 24267–24277.

Cho, H.S., Mason, K., Ramyar, K.X., Stanley, A.M., Gabelli, S.B., Denney, D.W., Jr., and Leahy, D.J. (2003). Structure of the extracellular region of HER2 alone and in complex with the Herceptin Fab. Nature *421*, 756–760.

Daemen, A., Griffith, O.L., Heiser, L.M., Wang, N.J., Enache, O.M., Sanborn, Z., Pepin, F., Durinck, S., Korkola, J.E., Griffith, M., et al. (2013). Modeling precision treatment of breast cancer. Genome Biol. *14*, R110.

Debnath, J., Muthuswamy, S.K., and Brugge, J.S. (2003). Morphogenesis and oncogenesis of MCF-10A mammary epithelial acini grown in three-dimensional basement membrane cultures. Methods *30*, 256–268.

DeNardo, D.G., Brennan, D.J., Rexhepaj, E., Ruffell, B., Shiao, S.L., Madden, S.F., Gallagher, W.M., Wadhwani, N., Keil, S.D., Junaid, S.A., et al. (2011). Leukocyte complexity predicts breast cancer survival and functionally regulates response to chemotherapy. Cancer Discov. *1*, 54–67.

Diéras, V., Miles, D., Verma, S., Pegram, M., Welslau, M., Baselga, J., Krop, I.E., Blackwell, K., Hoersch, S., Xu, J., et al. (2017). Trastuzumab emtansine versus capecitabine plus lapatinib in patients with previously treated HER2-positive advanced breast cancer (EMILIA): a descriptive analysis of final overall survival results from a randomised, open-label, phase 3 trial. Lancet Oncol. *18*, 732–742.

Diermeier-Daucher, S., Breindl, S., Buchholz, S., Ortmann, O., and Brockhoff, G. (2011). Modular anti-EGFR and anti-Her2 targeting of SK-BR-3 and BT474 breast cancer cell lines in the presence of ErbB receptor-specific growth factors. Cytometry A *79*, 684–693.

Donnelly, S.M., Paplomata, E., Peake, B.M., Sanabria, E., Chen, Z., and Nahta, R. (2014). P38 MAPK contributes to resistance and invasiveness of HER2-overexpressing breast cancer. Curr. Med. Chem. *21*, 501–510.

Eagles, G., Warn, A., Ball, R.Y., Baillie-Johnson, H., Arakaki, N., Daikuhara, Y., and Warn, R.M. (1996). Hepatocyte growth factor/scatter factor is present in most pleural effusion fluids from cancer patients. Br. J. Cancer *73*, 377–381.

Finigan, J.H., Faress, J.A., Wilkinson, E., Mishra, R.S., Nethery, D.E., Wyler, D., Shatat, M., Ware, L.B., Matthay, M.A., Mason, R., et al. (2011). Neuregulin-1-human epidermal receptor-2 signaling is a central regulator of pulmonary epithelial permeability and acute lung injury. J. Biol. Chem. *286*, 10660–10670.

Franklin, M.C., Carey, K.D., Vajdos, F.F., Leahy, D.J., de Vos, A.M., and Sliwkowski, M.X. (2004). Insights into ErbB signaling from the structure of the ErbB2-pertuzumab complex. Cancer Cell *5*, 317–328.

Gagnon-Bartsch, J.A., Jacob, L., and Speed, T.P. (2013). Removing Unwanted Variation from High Dimensional Data with Negative Controls (Department of Statistics, University of California, Berkeley), Report Number: 820.

Geyer, C.E., Forster, J., Lindquist, D., Chan, S., Romieu, C.G., Pienkowski, T., Jagiello-Gruszfeld, A., Crown, J., Chan, A., Kaufman, B., et al. (2006). Lapatinib plus capecitabine for HER2-positive advanced breast cancer. N. Engl. J. Med. *355*, 2733–2743.

Gomez, H.L., Doval, D.C., Chavez, M.A., Ang, P.C., Aziz, Z., Nag, S., Ng, C., Franco, S.X., Chow, L.W., Arbushites, M.C., et al. (2008). Efficacy and safety of lapatinib as first-line therapy for ErbB2-amplified locally advanced or metastatic breast cancer. J. Clin. Oncol. *26*, 2999–3005.

Gu, S., Hu, Z., Ngamcherdtrakul, W., Castro, D.J., Morry, J., Reda, M.M., Gray, J.W., and Yantasee, W. (2016). Therapeutic siRNA for drug-resistant HER2-positive breast cancer. Oncotarget *7*, 14727–14741.

Harbeck, N., Beckmann, M.W., Rody, A., Schneeweiss, A., Muller, V., Fehm, T., Marschner, N., Gluz, O., Schrader, I., Heinrich, G., et al. (2013). HER2 dimerization inhibitor pertuzumab - mode of action and clinical data in breast cancer. Breast Care (Basel) *8*, 49–55.

Hegde, G.V., de la Cruz, C.C., Chiu, C., Alag, N., Schaefer, G., Crocker, L., Ross, S., Goldenberg, D., Merchant, M., Tien, J., et al. (2013). Blocking NRG1 and other ligand-mediated Her4 signaling enhances the magnitude and duration of the chemotherapeutic response of non-small cell lung cancer. Sci. Transl. Med. *5*, 171ra18.

Heiser, L.M., Sadanandam, A., Kuo, W.L., Benz, S.C., Goldstein, T.C., Ng, S., Gibb, W.J., Wang, N.J., Ziyad, S., Tong, F., et al. (2012). Subtype and pathway specific responses to anticancer compounds in breast cancer. Proc. Natl. Acad. Sci. USA *109*, 2724–2729.

Huang, C., Park, C.C., Hilsenbeck, S.G., Ward, R., Rimawi, M.F., Wang, Y.C., Shou, J., Bissell, M.J., Osborne, C.K., and Schiff, R. (2011). beta1 integrin mediates an alternative survival pathway in breast cancer cells resistant to lapatinib. Breast Cancer Res. *13*, R84.

Johnston, S., Pippen, J., Jr., Pivot, X., Lichinitser, M., Sadeghi, S., Dieras, V., Gomez, H.L., Romieu, G., Manikhas, A., Kennedy, M.J., et al. (2009). Lapatinib combined with letrozole versus letrozole and placebo as first-line therapy for postmenopausal hormone receptor-positive metastatic breast cancer. J. Clin. Oncol. *27*, 5538–5546.

Jonas, O., Landry, H.M., Fuller, J.E., Santini, J.T., Jr., Baselga, J., Tepper, R.I., Cima, M.J., and Langer, R. (2015). An implantable microdevice to perform high-throughput in vivo drug sensitivity testing in tumors. Sci. Transl. Med. *7*, 284ra57.

Kamentsky, L., Jones, T.R., Fraser, A., Bray, M.A., Logan, D.J., Madden, K.L., Ljosa, V., Rueden, C., Eliceiri, K.W., and Carpenter, A.E. (2011). Improved structure, function and compatibility for CellProfiler: modular high-throughput image analysis software. Bioinformatics *27*, 1179–1180.

Kanda, T., Sullivan, K.F., and Wahl, G.M. (1998). Histone-GFP fusion protein enables sensitive analysis of chromosome dynamics in living mammalian cells. Curr. Biol. *8*, 377–385.

Kaufman, B., Trudeau, M., Awada, A., Blackwell, K., Bachelot, T., Salazar, V., DeSilvio, M., Westlund, R., Zaks, T., Spector, N., and Johnston, S. (2009). Lapatinib monotherapy in patients with HER2-overexpressing relapsed or refractory inflammatory breast cancer: final results and survival of the expanded HER2+ cohort in EGF103009, a phase II study. Lancet Oncol. *10*, 581–588.

Kodack, D.P., Askoxylakis, V., Ferraro, G.B., Sheng, Q., Badeaux, M., Goel, S., Qi, X., Shankaraiah, R., Cao, Z.A., Ramjiawan, R.R., et al. (2017). The brain microenvironment mediates resistance in luminal breast cancer to PI3K inhibition through HER3 activation. Sci. Transl. Med. *9*.

Korkola, J.E., Collisson, E.A., Heiser, L., Oates, C., Bayani, N., Itani, S., Esch, A., Thompson, W., Griffith, O.L., Wang, N.J., et al. (2015). Decoupling of the PI3K pathway via mutation necessitates combinatorial treatment in HER2+ breast cancer. PLoS One *10*, e0133219.

Kuo, W.L., Das, D., Ziyad, S., Bhattacharya, S., Gibb, W.J., Heiser, L.M., Sadanandam, A., Fontenay, G.V., Hu, Z., Wang, N.J., et al. (2009). A systems analysis of the chemosensitivity of breast cancer cells to the polyamine analogue PG-11047. BMC Med. *7*, 77.

Law, A.J., Shannon Weickert, C., Hyde, T.M., Kleinman, J.E., and Harrison, P.J. (2004). Neuregulin-1 (NRG-1) mRNA and protein in the adult human brain. Neuroscience *127*, 125–136.

Lee-Hoeflich, S.T., Crocker, L., Yao, E., Pham, T., Munroe, X., Hoeflich, K.P., Sliwkowski, M.X., and Stern, H.M. (2008). A central role for HER3 in HER2-amplified breast cancer: implications for targeted therapy. Cancer Res. *68*, 5878–5887.

Leung, W.Y., Roxanis, I., Sheldon, H., Buffa, F.M., Li, J.L., Harris, A.L., and Kong, A. (2015). Combining lapatinib and pertuzumab to overcome lapatinib resistance due to NRG1-mediated signalling in HER2-amplified breast cancer. Oncotarget *6*, 5678–5694.

Lin, C.H., Lee, J.K., and LaBarge, M.A. (2012). Fabrication and use of microenvironment microarrays (MEArrays). J. Vis. Exp. https://doi.org/10.3791/4152.

Lin, M.C., Rojas, K.S., Cerione, R.A., and Wilson, K.F. (2014). Identification of mTORC2 as a necessary component of HRG/ErbB2-dependent cellular transformation. Mol. Cancer Res. *12*, 940–952.

Moondra, V., Sarma, S., Buxton, T., Safa, R., Cote, G., Storer, T., Lebrasseur, N.K., and Sawyer, D.B. (2009). Serum Neuregulin-1beta as a biomarker of cardiovascular fitness. Open Biomark. J. *2*, 1–5.

Muranen, T., Selfors, L.M., Worster, D.T., Iwanicki, M.P., Song, L., Morales, F.C., Gao, S., Mills, G.B., and Brugge, J.S. (2012). Inhibition of PI3K/mTOR leads to adaptive resistance in matrix-attached cancer cells. Cancer Cell *21*, 227–239.

Ni, M., Chen, Y., Lim, E., Wimberly, H., Bailey, S.T., Imai, Y., Rimm, D.L., Liu, X.S., and Brown, M. (2011). Targeting androgen receptor in estrogen receptor-negative breast cancer. Cancer Cell *20*, 119–131.

Novotny, C.J., Pollari, S., Park, J.H., Lemmon, M.A., Shen, W., and Shokat, K.M. (2016). Overcoming resistance to HER2 inhibitors through state-specific kinase binding. Nat. Chem. Biol. *12*, 923–930.

Ryan, Q., Ibrahim, A., Cohen, M.H., Johnson, J., Ko, C.W., Sridhara, R., Justice, R., and Pazdur, R. (2008). FDA drug approval summary: lapatinib in combination with capecitabine for previously treated metastatic breast cancer that overexpresses HER-2. Oncologist *13*, 1114–1119.

Schindelin, J., Arganda-Carreras, I., Frise, E., Kaynig, V., Longair, M., Pietzsch, T., Preibisch, S., Rueden, C., Saalfeld, S., Schmid, B., et al. (2012). Fiji: an open-source platform for biological-image analysis. Nat. Methods *9*, 676–682.

Sergina, N.V., Rausch, M., Wang, D., Blair, J., Hann, B., Shokat, K.M., and Moasser, M.M. (2007). Escape from HER-family tyrosine kinase inhibitor therapy by the kinase-inactive HER3. Nature *445*, 437–441.

Slamon, D.J., Godolphin, W., Jones, L.A., Holt, J.A., Wong, S.G., Keith, D.E., Levin, W.J., Stuart, S.G., Udove, J., Ullrich, A., et al. (1989). Studies of the HER-2/neu proto-oncogene in human breast and ovarian cancer. Science *244*, 707–712.

Sorlie, T., Tibshirani, R., Parker, J., Hastie, T., Marron, J.S., Nobel, A., Deng, S., Johnsen, H., Pesich, R., Geisler, S., et al. (2003). Repeated observation of breast tumor subtypes in independent gene expression data sets. Proc. Natl. Acad. Sci. USA *100*, 8418–8423.

Straussman, R., Morikawa, T., Shee, K., Barzily-Rokni, M., Qian, Z.R., Du, J., Davis, A., Mongare, M.M., Gould, J., Frederick, D.T., et al. (2012). Tumour micro-environment elicits innate resistance to RAF inhibitors through HGF secretion. Nature *487*, 500–504.

Sullivan, R., Pare, G.C., Frederiksen, L.J., Semenza, G.L., and Graham, C.H. (2008). Hypoxia-induced resistance to anticancer drugs is associated with decreased senescence and requires hypoxia-inducible factor-1 activity. Mol. Cancer Ther. *7*, 1961–1973.

Takai, K., Hara, J., Matsumoto, K., Hosoi, G., Osugi, Y., Tawa, A., Okada, S., and Nakamura, T. (1997). Hepatocyte growth factor is constitutively produced by human bone marrow stromal cells and indirectly promotes hematopoiesis. Blood *89*, 1560–1565.

Thomas, G., Siegmann, M., and Gordon, J. (1979). Multiple phosphorylation of ribosomal protein S6 during transition of quiescent 3T3 cells into early G1, and cellular compartmentalization of the phosphate donor. Proc. Natl. Acad. Sci. USA *76*, 3952–3956.

Tibes, R., Qiu, Y., Lu, Y., Hennessy, B., Andreeff, M., Mills, G.B., and Kornblau, S.M. (2006). Reverse phase protein array: validation of a novel proteomic technology and utility for analysis of primary leukemia specimens and hematopoietic stem cells. Mol. Cancer Ther. *5*, 2512–2521.

Tiwari, S.R., Mishra, P., and Abraham, J. (2016). Neratinib, A novel HER2-targeted tyrosine kinase inhibitor. Clin. Breast Cancer *16*, 344–348.

Tyan, S.W., Kuo, W.H., Huang, C.K., Pan, C.C., Shew, J.Y., Chang, K.J., Lee, E.Y., and Lee, W.H. (2011). Breast cancer cells induce cancer-associated fibroblasts to secrete hepatocyte growth factor to enhance breast tumorigenesis. PLoS One *6*, e15313.

Uhlén, M., Fagerberg, L., Hallström, B.M., Lindskog, C., Oksvold, P., Mardinoglu, A., Sivertsson, Å., Kampf, C., Sjöstedt, E., Asplund, A., et al. (2015). Proteomics. Tissue-based map of the human proteome. Science *347*, 1260419.

Veenstra, C., Pérez-Tenorio, G., Stelling, A., Karlsson, E., Mirwani, S.M., Nordenskjöld, B., Fornander, T., and Stål, O. (2016). Met and its ligand HGF are associated with clinical outcome in breast cancer. Oncotarget *7*, 37145–37159.

Warnes, G.R., Bolker, B., Bonebakker, L., Gentleman, R., Liaw, W.H.A., Lumley, T., Maechler, M., Magnusson, A., Moeller, S., Schwartz, M., et al. (2016). Gplots: Various R Programming Tools for Plotting Data.

Weigelt, B., and Bissell, M.J. (2008). Unraveling the microenvironmental influences on the normal mammary gland and breast cancer. Semin. Cancer Biol. *18*, 311–321.

Wilson, T.R., Fridlyand, J., Yan, Y., Penuel, E., Burton, L., Chan, E., Peng, J., Lin, E., Wang, Y., Sosman, J., et al. (2012). Widespread potential for growth-factor-driven resistance to anticancer kinase inhibitors. Nature *487*, 505–509.

Wood, E.R., Truesdale, A.T., McDonald, O.B., Yuan, D., Hassell, A., Dickerson, S.H., Ellis, B., Pennisi, C., Horne, E., Lackey, K., et al. (2004). A unique structure for epidermal growth factor receptor bound to GW572016 (Lapatinib): relationships among protein conformation, inhibitor off-rate, and receptor activity in tumor cells. Cancer Res. *64*, 6652–6659.

Zhang, Q., Park, E., Kani, K., and Landgraf, R. (2012). Functional isolation of activated and unilaterally phosphorylated heterodimers of ERBB2 and ERBB3 as scaffolds in ligand-dependent signaling. Proc. Natl. Acad. Sci. USA *109*, 13237–13242.

STAR★METHODS

KEY RESOURCES TABLE

REAGENT or RESOURCE	SOURCE	IDENTIFIER
Antibodies		
Cytokeratin 14 (clone LL002)	Abcam	Cat#ab7800; RRID: AB_306091
Cytokeratin 19 (clone RSK108)	Dako	Cat#M088801-2; RRID: AB_2234418
HER2 (clone 29D8)	Cell Signaling	Cat#2615; RRID: AB_560966
HER2 (clone 3B5)	EMD Millipore	Cat#OP15F; RRID: AB_2246561
pHER2 Y1221/1222 (clone 6B12)	Cell Signaling	Cat#2243S; RRID: AB_490899
HER3 (clone D22C5)	Cell Signaling	Cat#12708S; RRID: N/A
pHER3 Y1298 (clone 21D3)	Cell Signaling	Cat#4791L; RRID: AB_2099708
panAKT (clone C67E7)	Cell Signaling	Cat#4691; RRID: AB_915783
pAKT S473 (clone D9E)	Cell Signaling	Cat#5012; RRID: AB_2224726
S6 (clone 54D2)	Cell Signaling	Cat#2317; RRID: AB_2238583
pS6 S235/236 (clone D57.2.2E)	Cell Signaling	Cat#4803; RRID: AB_916158
ERK1/2 (clone 137FS)	Cell Signaling	Cat#9101; RRID: AB_331646
pERK1/2 T202/Y204 (clone D13.14.4E)	Cell Signaling	Cat#4370; RRID: AB_2315112
Cleaved caspase 3	Cell Signaling	Cat#9661; RRID: AB_2341188
Ki67	Cell Signaling	Cat#12202; RRID: AB_2620142
IGF-1R	Cell Signaling	Cat#9750; RRID: AB_10950969
Tubulin beta-1	Santa Cruz	Cat#sc-9935 P; RRID: AB_2241172
Chemicals, Peptides, and Recombinant Proteins		
Lapatinib ditosylate HER2 inhibitor	Selleckchem	Cat#S1028
Neratinib HER2 inhibitor	Selleckchem	Cat#S2150
Trametinib MEK inhibitor	Selleckchem	Cat#S2673
Capecitabine MET inhibitor	Selleckchem	Cat#S1156
Crizotinib	Selleckchem	Cat#S1068
Pertuzumab	OHSU Pharmacy	NA
Trastuzumab	OHSU Pharmacy	NA
Human IgG isotype control	Abcam	Cat#ab206195
DMSO	ThermoFisher	Cat#20688
alamarBlue Cell Viability reagent	ThermoFisher	Cat#DAL1025
Matrigel	BD Biosciences	Cat#354234
Triton X-100	Sigma-Aldrich	Cat#X100-500ML
ProteaseMax	ProMega	Cat#V2071
Formic acid	ThermoFisher	Cat#28905
Halt protease inhibitor	ThermoFisher	Cat#78430
Nonidet-P40	ThermoFisher	Cat#28324
Dharmafect	Dharmacon	Cat#T-2001-01
DAPI FluoroPure grade	ThermoFisher	Cat#D21490
Goat anti-Mouse IgG3 Alexa Fluor 488	ThermoFisher	Cat#A-21151
Goat anti-Mouse IgG1 Alexa Fluor 555	ThermoFisher	Cat#A-21127
Polyethylene glycol MW1450	Polysciences	Cat#00679-250
Trypsin .25%	ThermoFisher	Cat#25200056
Recombinant human NRG1-β EGF domain	R&D Systems	Cat#396-HB-050/CF
Recombinant human HGF	R&D Systems	Cat#294-HG-005/CF
*All other MEMA protein information available online	Synapse	https://www.synapse.org/#!Synapse:syn2874083.3

(*Continued on next page*)

Continued		
REAGENT or RESOURCE	SOURCE	IDENTIFIER
Critical Commercial Assays		
Duolink Proximity Ligation Assay	Sigma-Aldrich	Cat#DUO92101
Click-iT EdU Alexa Fluor 647 Imaging Kit	ThermoFisher	Cat#C10640
BCA protein assay reagent	ThermoFisher	Cat#23225
Deposited Data		
MicroEnvironment MicroArray data for AU565 and HCC1954 control and lapatinib treatments	This paper	https://www.synapse.org/#!Synapse:syn7876903
RNAseq of breast cancer cell lines	Daemen et al., 2013	GEO: GSE48213
RPPA of breast cancer cell lines	Korkola et al., 2015	https://www.synapse.org/#!Synapse:syn2346643/wiki/232048
Experimental Models: Cell Lines		
Human: AU565	ATCC	RRID: CVCL_1074
Human: SKBR3	ATCC	RRID: CVCL_0033
Human: SKBR3-NucGFP	Kanda et al., 1998	NA
Human: BT474	ATCC	RRID: CVCL_0179
Human: BT474-TRgf	Robert Kerbel, Giulio Francia	NA
Human: EFM192A	DSMZ	RRID: CVCL_1812
Human: EFM192B	DSMZ	RRID: CVCL_1813
Human: EFM192C	DSMZ	RRID: CVCL_1814
Human: ZR-75-30	ATCC	RRID: CVCL_1661
Human: SUM190PT	Steve Ethier	RRID: CVCL_3423
Human: SUM225CWN	Steve Ethier	RRID: CVCL_5593
Human: HCC-202	ATCC	RRID: CVCL_2062
Human: HCC-1419	ATCC	RRID: CVCL_1251
Human: HCC-1569	ATCC	RRID: CVCL_1255
Human: HCC-1954	ATCC	RRID: CVCL_1259
Human: HCC-1954-NucGFP	Kanda et al., 1998	NA
Human: HCC-2218	ATCC	RRID: CVCL_1263
Human: HCC-3153	UT-Southwestern	RRID: CVCL_3377
Human: 21NT1	Kornelia Polyak, Ruth Sager	NA
Human: 21PT1	Kornelia Polyak, Ruth Sager	NA
Human: 21MT1	Kornelia Polyak, Ruth Sager	RRID: CVCL_7931
Human: JIMT1	DSMZ	RRID: CVCL_2077
Human: MDA-MB-361	ATCC	RRID: CVCL_0620
Human: MDA-MB-453	ATCC	RRID: CVCL_0418
Human: UACC-893	ATCC	RRID: CVCL_1782
Experimental Models: Organisms/Strains		
Mouse: SCID, SHO-*Prkdc*^{scid}*Hr*^{hr}	Charles River Laboratories	Strain Code: 474
Mouse: NU(NCr)-Foxn1^{nu}	Charles River Laboratories	Strain Code: 490
Oligonucleotides		
HER3 siRNA J-003127-10	Dharmacon	Cat#J-003127-10-0002
HER3 siRNA J-003127-11	Dharmacon	Cat#J-003127-11-0002
HER3 siRNA J-003127-12	Dharmacon	Cat#J-003127-12-0002
HER3 siRNA J-003127-13	Dharmacon	Cat#J-003127-13-0002
ON-TARGETplus Non-targeting siRNA	Dharmacon	Cat#D-0008180-01-05

(*Continued on next page*)

Continued		
REAGENT or RESOURCE	SOURCE	IDENTIFIER
Software and Algorithms		
CellProfiler	Kamentsky et al., 2011	http://cellprofiler.org/
Gene Set Enrichment Analysis	Subramanian, Tamayo, et al.	http://software.broadinstitute.org/gsea/index.jsp
gplots	Warnes et al., 2016	https://CRAN.R-project.org/package=gplots
QI Systems	Nederlof and Sudar, 2016	http://www.qi-tissue.com/
Fiji	Schindelin et al., 2012	

CONTACT FOR REAGENT AND RESOURCE SHARING

Further information and requests for resources and reagents should be directed to and will be fulfilled by the lead contact, James E. Korkola (korkola@ohsu.edu).

EXPERIMENTAL MODELS AND SUBJECT DETAILS

Breast Cancer Cell Lines

Breast cancer cell lines derived from human female tumors were used in this study. The cell lines AU565, SKBR3, HCC1954, HCC-1569, HCC-202, HCC-2218, HCC-1419, MDA-MB-361, ZR-75-30, BT474, UACC893, and MDA-MB-453 were obtained from American Type Culture Collection (ATCC), Manassas, VA. HCC-3153 was obtained from UT-Southwestern, SUM190PT and SUM225CWN were provided by Steve Ethier at UCSF, and 21NT1, 21PT1, and 21MT1 were provided by Ruth Sager and Kornelia Polyak at the Dana-Farber Institute of Harvard, Cambridge MA. JIMT1, EFM192A, EFM192B, and EFM192C were obtained from DSMZ, Braunschweig Germany. Each cell line was genotyped to ensure accurate identity, and regularly screened for mycoplasma infection. Cell lines were maintained in their respective medium and serum concentration as recommended by originator specifications at 37°C in 5% CO2 in a humidified incubator and cultured according to ATCC recommendations.

Murine Models

SCID, SHO-*Prkdc*^{scid}*Hr*^{hr} and NU(NCr)-Foxn1^{nu} mice were purchased from Charles River Laboratories. All animal studies were conducted in accordance with protocols approved by MIT's Committee on Animal Care (CAC) and by Institutional Animal Care and Use Committee (IACUC) at OHSU.

METHOD DETAILS

Experimental Design

The number of independent biological replicates of each experiment (n) performed are given in the figure legends. Where appropriate the mean and standard error of the mean (SEM) were calculated as indicated. There was no blinding of any experimental data, and no sample-size estimation or randomization were used in standard drug treatment experiments. Protein combination printing locations in MEMA experiments, and drug treatment plates in Figure 4F were randomized. Experimental results were reproduced in at least three technical replicates (TR), and included either n=3 sample replicates, or were reproduced with at least 3 biological replicates (BR). No data were excluded from published results, except for cell count and proliferation ratio of spots containing nidogen in Figure 1. These data were omitted from Figures 1B and 1C as outliers (as noted in figure legend).

Drug Treatment

Lapatinib, neratinib, crizotinib, trametinib, capecitabine (Selleckchem), pertuzumab, and trastuzumab (OHSU Pharmacy) were used at the concentrations indicated in figure legends. DMSO (ThermoFisher) and human IgG isotype control (Abcam) concentrations were equivalent to the highest dose of the respective drug used in each experiment. Treatment durations were as indicated in respective figure legends. Cells were treated in 96 and 384 multiwell plates with soluble drug and ligand combinations added to their medium, then fixed for fluorescent imaging and quantification (described below). Each treated cell line was seeded at an experimentally determined concertation so that untreated control wells would reach 80% confluency by the end of the treatment period. Drug combination studies and CTG assays in Figure 4F were performed as previously reported (Heiser et al., 2012) (Kuo et al., 2009) in randomized replicates.

Fluorescence Cell Line Generation

SKBR3 and HCC1954 cell lines expressing nuclear localized GFP has been previously described (Kanda et al., 1998). Cell lines were maintained in their respective medium as recommended by ATCC at 37°C in 5% CO2 in a humidified incubator and cultured according to ATCC recommendations.

MicroEnvironment MicroArrays

MEMAs were generated in 8-well cell culture plates. A manuscript detailing the preparation and use of the MEMA is underway. A detailed description of the methodology and a list of the ECM components, soluble ligands, and their concentrations is currently available at the Synapse MEP-LINCs website (https://www.synapse.org/#!Synapse:syn2862345/wiki/72486). Proteins on the MEMA were chosen because of their reported involvement at sites of local and metastatic disease, and their capacity to elicit a biological effect in *in vitro* assays. The proteins included in the library represent components of lymphocytic infiltrates, stroma, blood and lymphatic system, local extracellular matrix, macrophages, and endothelium. Each matrix protein was mixed with collagen I to improve printing and cell attachment, and printed in ~15 replicate random locations (Figure S1A). We added soluble ligand to the wells, so an entire MEMA experiment comprised eight plates (seven ligands plus a PBS control well per plate; thus, 8 plates comprised all 56 ligands tested). $2.5x10^5$ cells of each cell line were added to replicate arrays for 15 minutes, after which unbound cells were removed with a growth medium wash. Arrays were cultured in RPMI medium with 10% fetal bovine serum for 12 hours at 37°C in 5% CO2 in a humidified incubator. Following this, appropriate concentrations of soluble ligands were added to duplicate sets of arrays. One set of arrays were treated with 750 nm lapatinib, and the other DMSO. Arrays were returned to incubator for 71 hours, after which 1uM EdU was added to the medium for 1 hour. Cells were then fixed in 2% PFA at RT, and stored at 4°C in PBS.

After fixation, EdU detection and immunofluorescent histochemistry (IHC) was performed as described below. Arrays were imaged on a customized automated high content fluorescence microscope platform (Nikon HCA), and resulting image data was output to an OMERO image database (Allan et al., 2012). Cells were segmented and intensity levels were calculated using CellProfiler (Kamentsky et al., 2011). The resulting MEMA data was preprocessed and normalized using open source R software available from (https://www.synapse.org/#!Synapse:syn2862345/wiki/72486). The spot cell count was based on the DAPI stained nuclei. EdU intensity was auto-gated to label cells as EdU^+ and the proportion of EdU^+ cells in each spot was reported to measure proliferation. The per-cell intensity values for the KRT14 and KRT19 stains and the nuclear morphology measurements were median summarized to the spot level. Each intensity and morphology signal was independently RUV normalized in a series of matrices with arrays as the rows and spots as the columns (Gagnon-Bartsch et al., 2013). The RUV controls were the residuals created by subtracting the replicate median from each spot value. After RUV normalization, bivariate LOESS normalization was applied to the normalized residuals using the array row and array column as the independent variables. After normalization, the ~15 replicates of each condition were median summarized to the MEP level. Major findings from the MEMA were recapitulated in at least 3 experimental replicates. Exact replicate count and standard error for each condition are available in supplemental MEMA files linked to in Data Availability.

Immunofluorescent Histochemistry and Fluorescent Imaging

Array-bound and well-bound cells were fixed in 2% PFA for 15 minutes at RT following respective treatments. Cells were then permeabilized with .3% Triton X-100 for 25 minutes at RT. Array-bound cell primary antibody staining was performed with KRT14 (Abcam, 1:200), KRT19 (Dako, 1:200), and DAPI (ThermoFisher, 1:10,000). Secondary antibody staining was performed with IgG3 Alexa Fluor 488 (ThermoFisher, 1:200), and IgG1 Alexa Fluor 555 (ThermoFisher, 1:200). Only DAPI and EdU detection was performed on well-bound cells, with the exception of Figure 2B. Well plates were imaged on the GE InCell 6000 platform, and image analysis and cell count quantification were performed on the GE InCell Analyzer software package. Size gating of nuclei was used to exclude apoptotic cells, and EdU positivity was determined as nuclei having a mean fluorescent intensity above an experimentally consistent threshold (this threshold was defined using single cell parametric analysis plotting total DAPI intensity against mean EdU intensity). All fluorescent imaging studies were performed at consistent intensity and gain settings across experiments.

EdU Incorporation

Cells were incubated with 1 μM EdU for 1 h prior to fixation. Cells were fixed, permeabilized, and stained with Click-iT Plus EdU Alexa Fluor 647 HCS Assay Kit (ThermoFisher) following manufacturers recommended protocol.

Live Cell Imaging

Live-cell imaging experiments were performed on the IncuCyte ZOOM platform with SKBR3 and HCC1954 cells transfected with a nuclear located GFP. Cell cohorts exposed to varying concentrations of NRG1β or HGF had these factors added to their medium at time zero of the time course. At 24 hours from the start of the experiment cells were exposed to requisite doses of lapatinib. Cells were fluorescently imaged every 2 hours (4 images per well), and Incucyte proprietary image analysis software quantified detected nuclei (following size gating to exclude apoptotic bodies and un-segmentable clusters). Concentrations were as noted in figure legends of Figures 1D, 1E, and S3. Live-cell time course experiments had n=2 biological replicates in each experiment, and all had n=3 technical replicates with consistent results.

3D Cultures

3D assays were performed using a previously described approach of coating well plates with matrigel matrix (Corning), plating cells, and adding medium with low density matrigel (Debnath et al., 2003). Cell quantity was assessed using absorbance measurements of alamar blue stains.

Proximity Ligation Assays

We performed PLA to detect the interactions between the c-terminal domains of HER2 and HER3 with the Duolink PLA kit (Sigma-Aldrich) according to the manufacturer's recommendations with at least 2 biological replicates per sample, and 3 technical replicates. Cells were exposed to growth factors and drug combinations as previously described, then fixed in 4% PFA, permeabilized with Triton X-100, and the Duolink PLA protocol was followed using HER2 (clone 3B5) and HER3 (clone D22C5) antibodies purchased from Cell Signaling Technology. Because of the abundance of HER2-HER3 heterodimers in SKBR3 cells, the assay was slightly modified to reduce detection of total HER2-HER3 dimers for the purpose of more accurate quantification. HER2-HER3 heterodimers were detected as single fluorescent dots in z-series of cells imaged with confocal microscopy. Additionally, cell nuclei were fluorescently stained with DAPI, and cellular cytoskeletons were labeled with tubulin antibody staining. The image analysis software CellProfiler (Kamentsky et al., 2011) was used to quantify the PLA signal.

Liposomal siRNA Transfection

siRNA transfection of breast cancer cell lines in 96-well plates (AU565 7,000/well, SKBR3 7,000/well, BT474 7000/well, HCC1954 4000/well, JIMT1 2000/well, 21MT1 1000/well, and HCC3153 2000/well) was performed by reverse transfection by using Dharmafect (Dharmacon) as previously described (Lee-Hoeflich et al., 2008). Four single siRNA oligos (Dharmacon HER3 J-003127-10, J-003127-11, J-003127-12, and J-003127-13; 12 nM each) were used for HER3, and non-targeting siRNA (Dharmacon siCONTROL) was used as a control. Following 96 hours of treatment with siRNA, cells were assayed for viable cell count as described above, and the average cell counts resulting from treatment with the 4 HER3 oligos were reported as comparisons to non-targeting siRNA control.

Protein Expression by RPPA or Immunoblots

RPPA and analysis were performed as previously described (Tibes et al., 2006) on cell lysates obtained from HCC1954 and AU565 cells treated for 0.5, 2, 4, 8, 24, 48, and 72 hours with 250 nM lapatinib in full serum medium. For Western blots, cell lysates were collected using Nonidet-P40 lysis buffer supplemented with Halt protease and phosphatase inhibitor cocktail (Thermo Scientific) and immunodetection of proteins was carried out using standard protocols for equal amounts of protein loaded in SDS gels (as determined by BCA protein abundance assays). The antibodies HER2 (clone 29D8), pHER2 (Y1221/1222, clone 6B12), HER3 (clone D22C5), pHER3 (Y1298, clone 21D3), panAKT (clone C67E7), pAKT (S473, clone D9E), S6 (clone 54D2), pS6 (S235/236, clone D57.2.2E), ERK1/2 (clone 137FS), and pERK1/2 (T202/Y204, clone D13.14.4E) were all purchased from Cell Signaling Technologies. Immunoblots were imaged on the LI-COR Odyssey platform, and quantified using LI-COR Image Studio Lite.

Immunoprecipitation Mass Spectrometry

In vitro cell culture treatments of DMSO, human IgG isotype control, NRG1β, lapatinib and pertuzumab were performed as described previously. Immunoprecipitated fractions from whole cell lysate were applied to NuPAGE 10% Bis-Tris SDS-PAGE gels (NP0301BOX), electrophoresed for 6 min at 200 V to remove impurities, and stained for 30 min with Imperial Blue protein stain (purchased from Thermo Scientific) to assess sample concentration and quality. Gels were washed in water and the entire top of each lane, from the bottom of the loading well to the tracking dye, was excised. Gel slices were then cut into 1 mm pieces, processed, reduced/alkylated, and digested with trypsin for one hour at 50°C in the presence of 0.01% ProteaseMax detergent (ProMega) using the method recommended from the manufacturer. Recovered peptides were then dried by vacuum centrifugation then dissolved in 5% formic acid in preparation for LC/MS analysis.

Digests were loaded onto an Acclaim PepMap 0.1 x 20 mm NanoViper C18 peptide trap (Thermo Scientific) for 5 min at a 5 μl/min flow rate in a 0.1% formic acid mobile phase. Peptides were then separated using a PepMap RSLC C18, 2 μm particle, 75 μm x 25 cm EasySpray column (Thermo Scientific) and 7.5–30% acetonitrile gradient over 60 min in mobile phase containing 0.1% formic acid at a 300 nl/min flow rate using a Dionex NCS-3500RS UltiMate RSLCnano UPLC system. Tandem mass spectrometry data was collected using an Orbitrap Fusion Tribrid mass spectrometer configured with an EasySpray NanoSource (Thermo Scientific). Survey scans were performed in the Orbitrap mass analyzer at 120,000 resolution, and data-dependent MS2 scans in the linear ion trap using HCD following isolation with the instrument's quadrupole.

Sequest (version 28, revision 12; Thermo Scientific) was used to search MS2 spectra against a June 2016 version of the Sprot human FASTA protein database, with added concatenated sequence-reversed entries to estimate error thresholds, and 179 common contaminant sequences and their reversed forms. The database processing was performed with Python scripts that have been described previously 1. Searches for all samples were performed with trypsin enzyme specificity. The monoisotopic parent and fragment ion mass tolerances were 1.25 and 1.0 Da, respectively. A static modification of +57.02 Da was added to all cysteine residues. A variable modification of +16 Da on methionine residues was also allowed, with a maximum of 3 modifications per peptide. A linear discriminant transformation was used to improve the identification sensitivity from the SEQUEST analysis 1,2. SEQUEST scores were combined into linear discriminant function scores, and discriminant score histograms were created separately for each peptide charge state (1+, 2+, and 3+). Separate histograms were created for matches to forward sequences and for matches to reversed sequences for all peptides of 7 amino acids or longer. Scores of histograms for reversed matches were used to estimate peptide false-discovery rates (FDR) and set score thresholds for each peptide class and a minimum of at least two unique peptide assignments to a protein entry was required across samples. This achieved a final protein FDR of 1.1%.

RNAseq

RNAseq analysis of purified mRNA from cell lysate was performed by our group as previously described (Daemen et al., 2013).

Murine Model Experiments

Murine Xenograft Implantable Microdevices

Microdose drug delivery devices were manufactured and implanted as previously described (Jonas et al., 2015). Cylindrical micro-devices 4mm in length and 820 μm in diameter were manufactured from medical-grade Delrin acetyl resin blocks (DuPont) by micro-machining (CNC Micromachining Center) with 18 reservoirs 200 μm (diameter) x 250 μm (depth) on the outer surface. Reservoirs were packed with approximately 1 μg of drug mixed with Polyethylene glycol (PEG, MW 1450, Polysciences) polymer using a tapered metal needle (Electron Microscopy Science). Lyophilized growth factors and proteins were packed on top of the drug mixture at approximately 5-10% of the reservoir volume. Pure PEG was used in control conditions.

Devices were implanted into orthotopic BT474 and subcutaneous JIMT1 xenograft tumors of three 6-8 week old female NOD SCID and Nu/Nu mice, respectively (purchased from Charles River Laboratories). Tumors were excised 48 hours after device implantation, fixed for 24 hours in 10% formalin, then perfused with paraffin. Specimens were sectioned using a standard microtome and sections were collected from each reservoir. Sections were then antibody stained by standard IHC using cleaved caspase-3 (9661, Cell Signaling Technology, CST) and Ki67 (12202, CST) antibody.

Additional Methods for BT474 Xenograft Experiments

100 μl of BT474 cells plus matrigel (BD Biosciences 354234) went into each site. BT474-TRgf (resistant to trastuzumab in vivo) was from Drs. Robert Kerbel (University of Toronto) and Giulio Francia (now at University of Texas at El Paso). Mice were bred from the Transgenic Core at OHSU.

QUANTIFICATION AND STATISTICAL ANALYSIS

Information on biological replicates (indicated as n) and technical replicates (indicated as TR) can be found in the respective figure legends. The reported statistics used sample means, standard error of the mean (SEM), and p-values obtained from unpaired para-metric t-tests of sample sizes of equivalent variance (unless otherwise noted in figure legends). All reported cell assays had at least 3 technical replicates, and 3 biological replicates (unless otherwise noted in figure legends).

RNAseq Hierarchal Clustering

Clustering analysis of RNAseq data was performed using open source R statistical software and the 'gplots' library. Genes deter-mined by TCGA (Cancer Genome Atlas Network, 2012) to be differentially expressed between patient tumors identified as HER2+ and expressing the HER2E PAM50 gene signature, and those identified as HER2+ but lacking the HER2E signature, were used to cluster RNAseq data obtained from a panel of human HER2+ breast cancer cell lines (Daemen et al., 2013). The differentially expressed gene list from patient data was filtered for genes expressed in the HER2+ breast cancer cell lines in the panel. Gene expression variance was determined using R, and the top 10% variable of the gene set were used to cluster the cell lines by Euclidean distance.

Gene Set Enrichment Analysis

Unbiased GSEA comparisons were performed between 8 L-HER2+ cell lines (AU565, BT474, SKBR3, ZR-75-30, UACC812, EFM192A, EFM192B, EFM192C), and 8 HER2E cell lines (JIMT1, 21MT1, 21MT2, 21NT, 21PT, HCC1569, HCC1954, HCC3153) using the javaGSEA Desktop Application available from the Broad Institute

https://CRAN.R-project.org/package=gplots

(http://software.broadinstitute.org/gsea/index.jsp). Gene sets with nominal p-values of less than 0.001, and false discovery rate q-values of less than 25% were considered significantly enriched.

DATA AND SOFTWARE AVAILABILITY

MicroEnvironment MicroArray Data

All MEMA data for the AU565 and HCC1954 cells is available via from the LINCS data portal (http://lincsportal.ccs.miami.edu/datasets-beta/#?query=assayname:MEMA cell growth assay) under accession numbers LDS-1467, LDS-1471, LDS-1475, and LDS-1479. All original image data is viewable on: https://omero.lincsclarion.org/webclient/?show=screen-251.

Immunoblot Supplement

A supplemental file showing full the full set of immunoblots is available as Data S1 (related to Figures 4, S5, and S6)

Cell Systems, Volume *6*

Supplemental Information

Microenvironment-Mediated Mechanisms

of Resistance to HER2 Inhibitors

Differ between HER2+ Breast Cancer Subtypes

Spencer S. Watson, Mark Dane, Koei Chin, Zuzana Tatarova, Moqing Liu, Tiera Liby, Wallace Thompson, Rebecca Smith, Michel Nederlof, Elmar Bucher, David Kilburn, Matthew Whitman, Damir Sudar, Gordon B. Mills, Laura M. Heiser, Oliver Jonas, Joe W. Gray, and James E. Korkola

Supplemental Figure S1

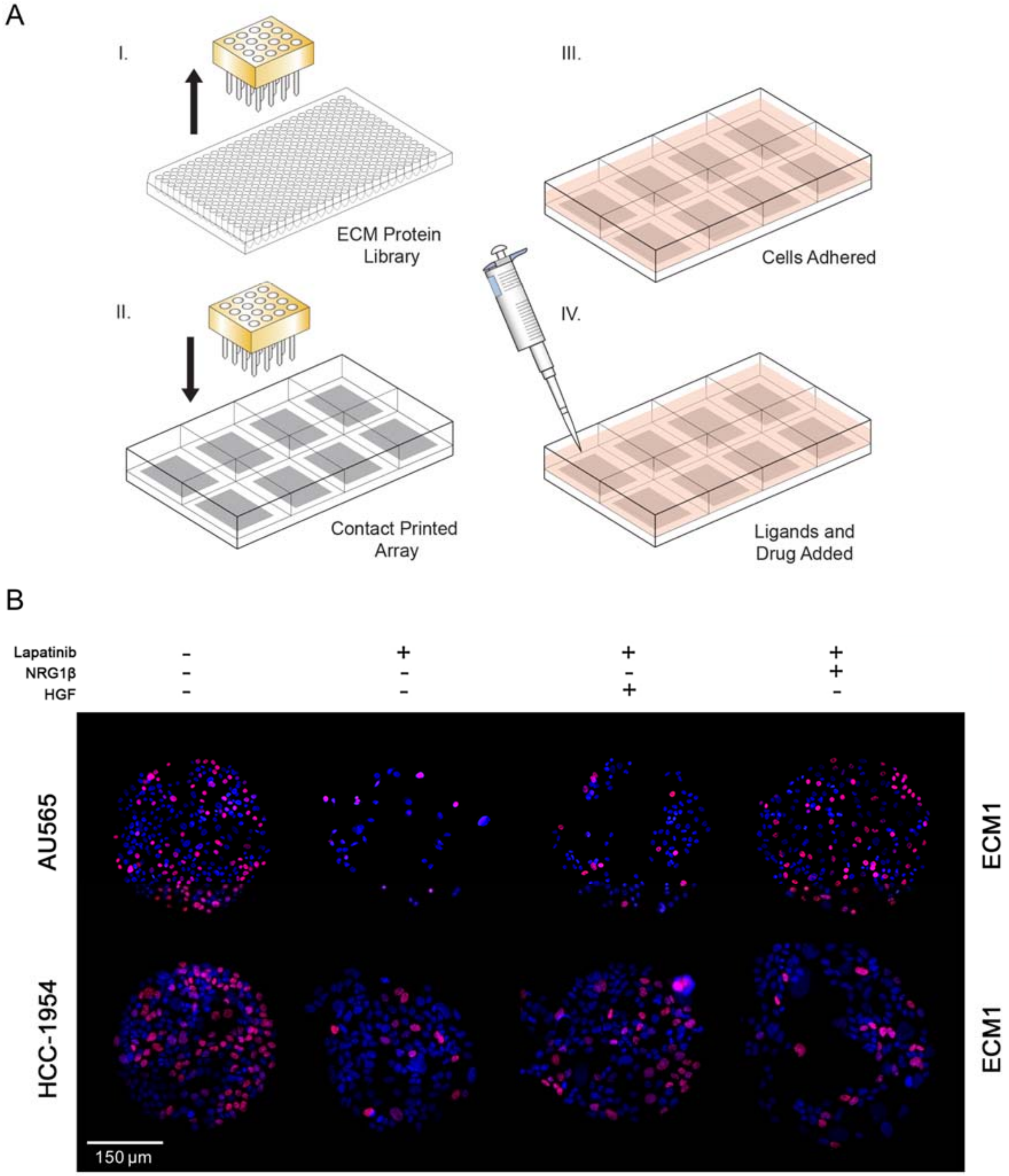

Supplemental Figure S1. Related to Figure 1 and STAR Methods. Schematic of MEMA preparation and representative images of cells grown on MEMA spots. (**a**) Libraries of biologically reactive human ECM and cell adhesion proteins immobilized onto a solid surface by randomized robotic contact printing. Cells adhered to printed protein spots, and exposed to a library of soluble functionalized human ligand proteins and 750 nM lapatinib or DMSO control. (**b**) Composite images of AU565 and HCC1954 cells on MEMA spots containing immobilized ECM1 protein, treated with combinations of lapatinib, NRG1β, and HGF. Nuclei labeled with DAPI (blue), proliferation measured by nuclear EdU uptake (pink).

Supplemental Figure S2

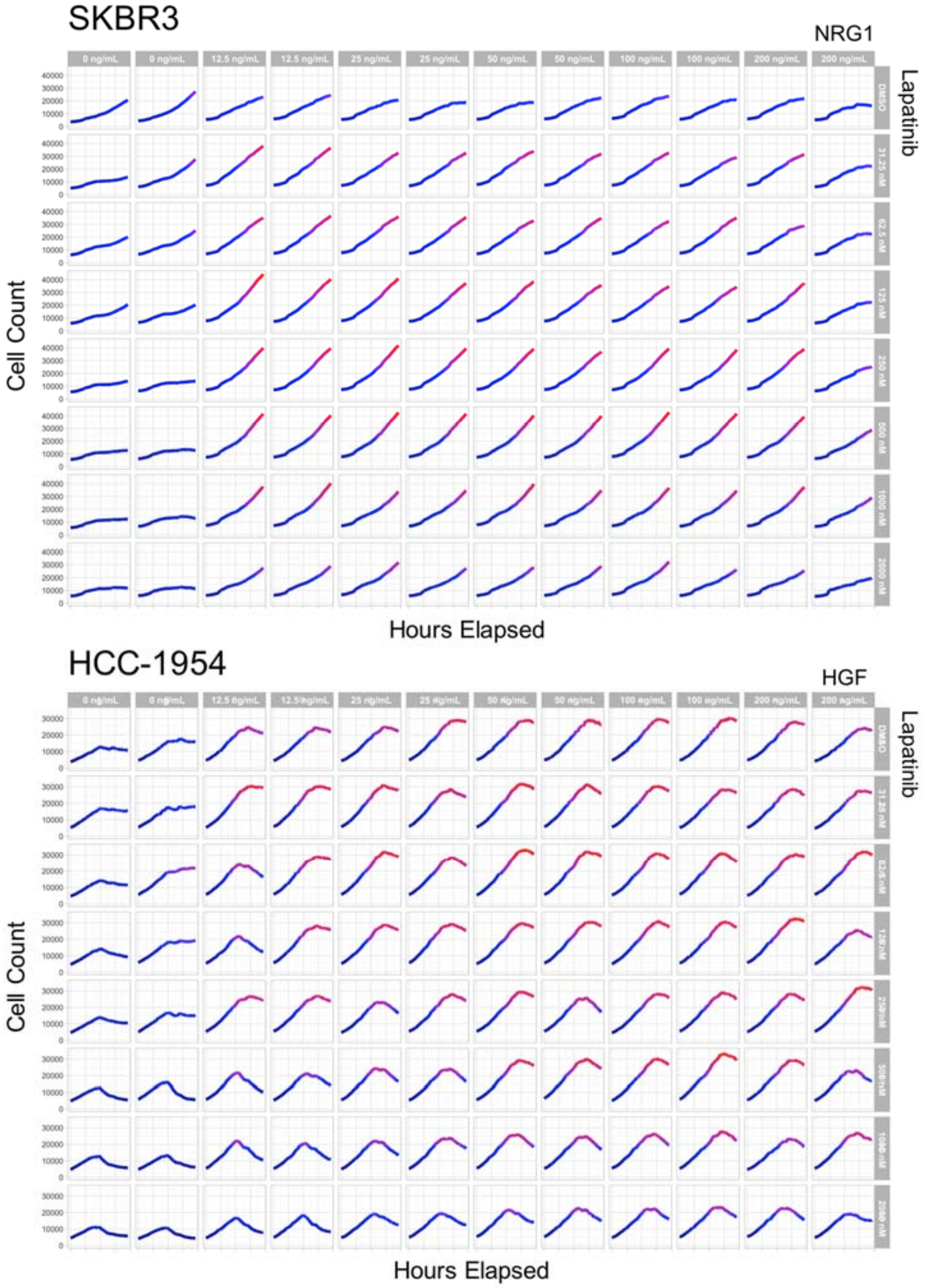

Supplemental Figure S2. Related to Figure 1. Live-cell Imaging reveals a range of concentrations of lapatinib and NRG1β or HGF that either restore proliferation, or enhance it over DMSO treated controls. Line graphs of cell count over 96 hours taken every 2 hours for a dose range of lapatinib versus a concentration range of NRG1β (for SKBR3), or HGF (for HCC1954). Concentration range for both ligands: 12.5, 25, 50, 100, and 200 ng/mL. Cohorts exposed to NRG1β or HGF had ligand added from the start of the experiment. For drug treated cohorts in both cell lines lapatinib was added 24 hours from the start of the experiment. Concentration range for lapatinib: 31.25, 62.5, 125, 250, 500, 1000, and 2000 nM. Red indicates cell count in excess of untreated controls.

Supplemental Figure S3

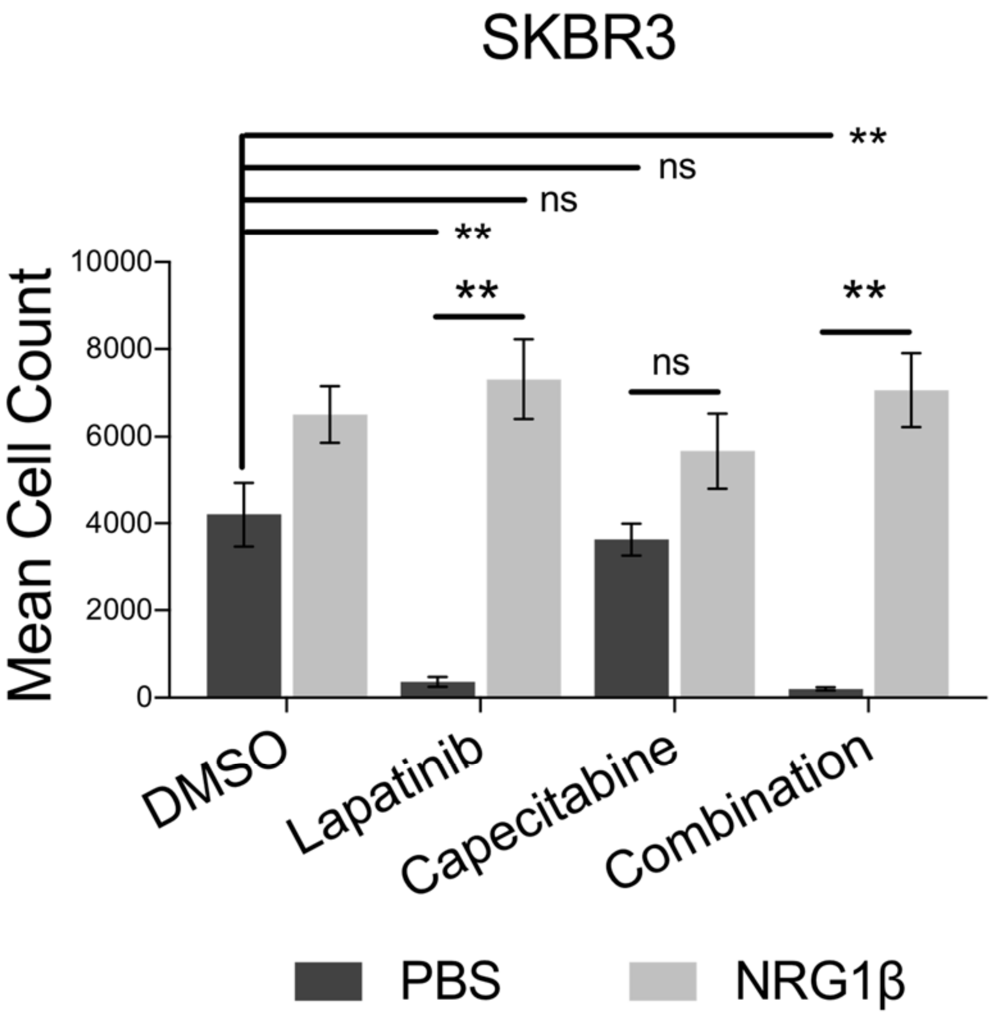

Supplemental Figure S3. Related to Figure 1. NRG1β drives resistance to combination treatment with lapatinib and capecitabine. Mean cell count and SEM (n = 3) for SKBR3 cells treated for 72 hours with combinations of DMSO, 500 nM lapatinib, 500 uM capecitabine, and 50 ng/mL NRG1β or HGF. Lapatinib significantly decreased cell counts compared to DMSO (p=0.0067), and addition of ligand significantly increased cell count compared to lapatinib alone (p=0.0016). Capecitabine decreased cell count compared to DMSO (ns.), but addition of ligand did not significantly increase cell count compared to capecitabine alone. The combination of lapatinib and capecitabine significantly decreased cell counts compared to DMSO (p=0.0056), and the addition of ligand significantly increased cell counts compared to combination (p=0.0012). Lapatinib plus ligand resulted in increased cell count compared to DMSO (ns). DMSO concentrations were matched to capecitabine concentrations.

Supplemental Figure S4

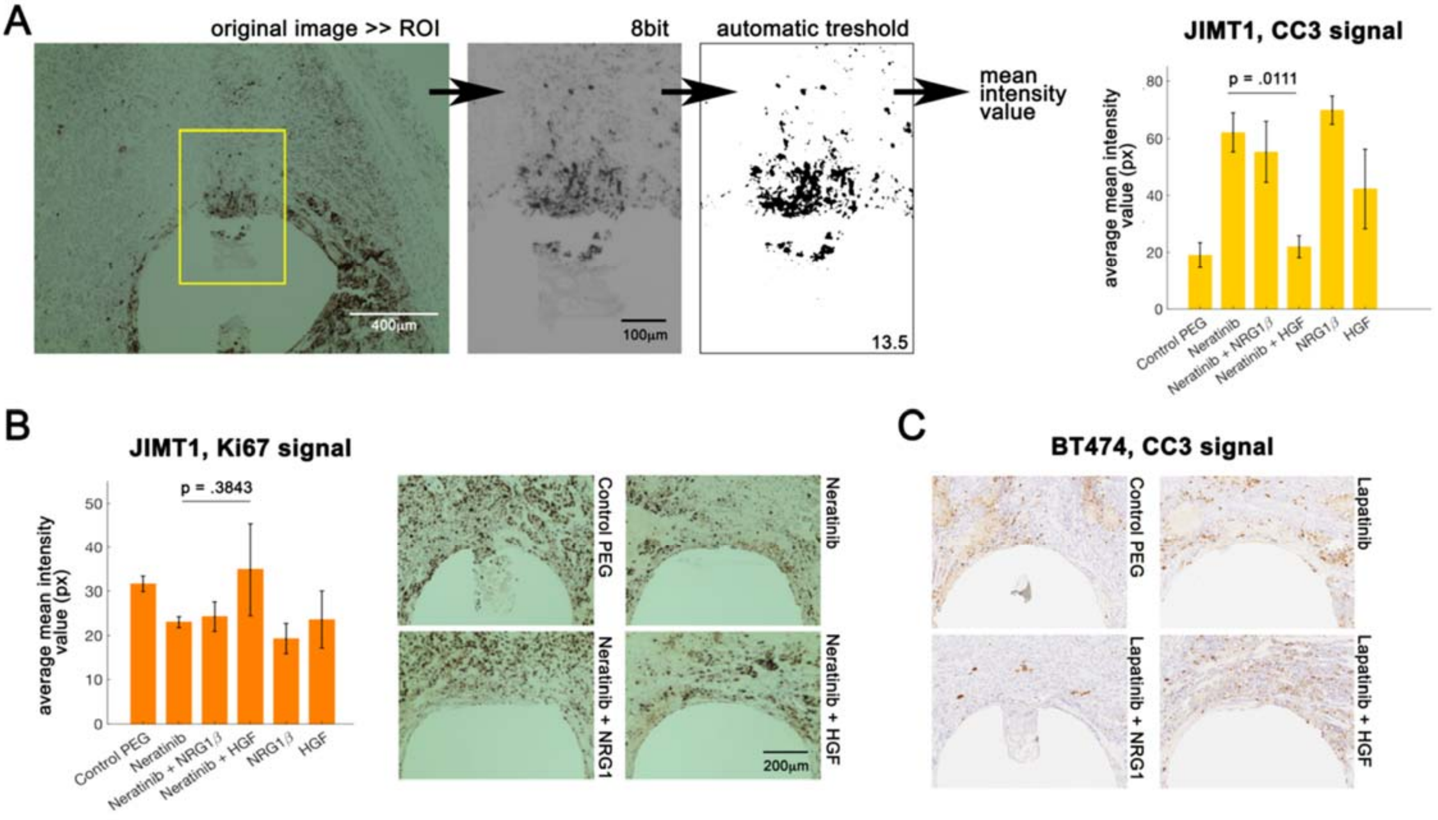

Supplemental Figure S4. Related to Figure 3. NRG1β and HGF mediated resistance to HER2 inhibition in vivo. (**a**) Schematic overview of the semiautomatic signal analysis using ImageJ (Schindelin et al., 2012) Briefly, region of interest was selected with the center at the drug release point. Cropped image was transformed to 8bit and automatic thresholding was applied. Mean intensity was measured and averaged to generate the graphs (right). (**b**) Addition of HGF to Neratinib restored the proliferation to control levels, however, the differences were not significant as compared to the single agent. Quantification (left) and supporting images of Ki67 stained JIMT1 tumor sections in close proximity to the reservoirs are shown (right). (**c**) Basal levels of apoptosis were minor in BT474 xenograft model and thus the effect of different conditions was compared by evaluating the Ki67 signal only (Figure 3E). Values in the graph are means and SEM (n = 3); significance was calculated by a paired sample two-tailed t-test.

Supplemental Figure S5

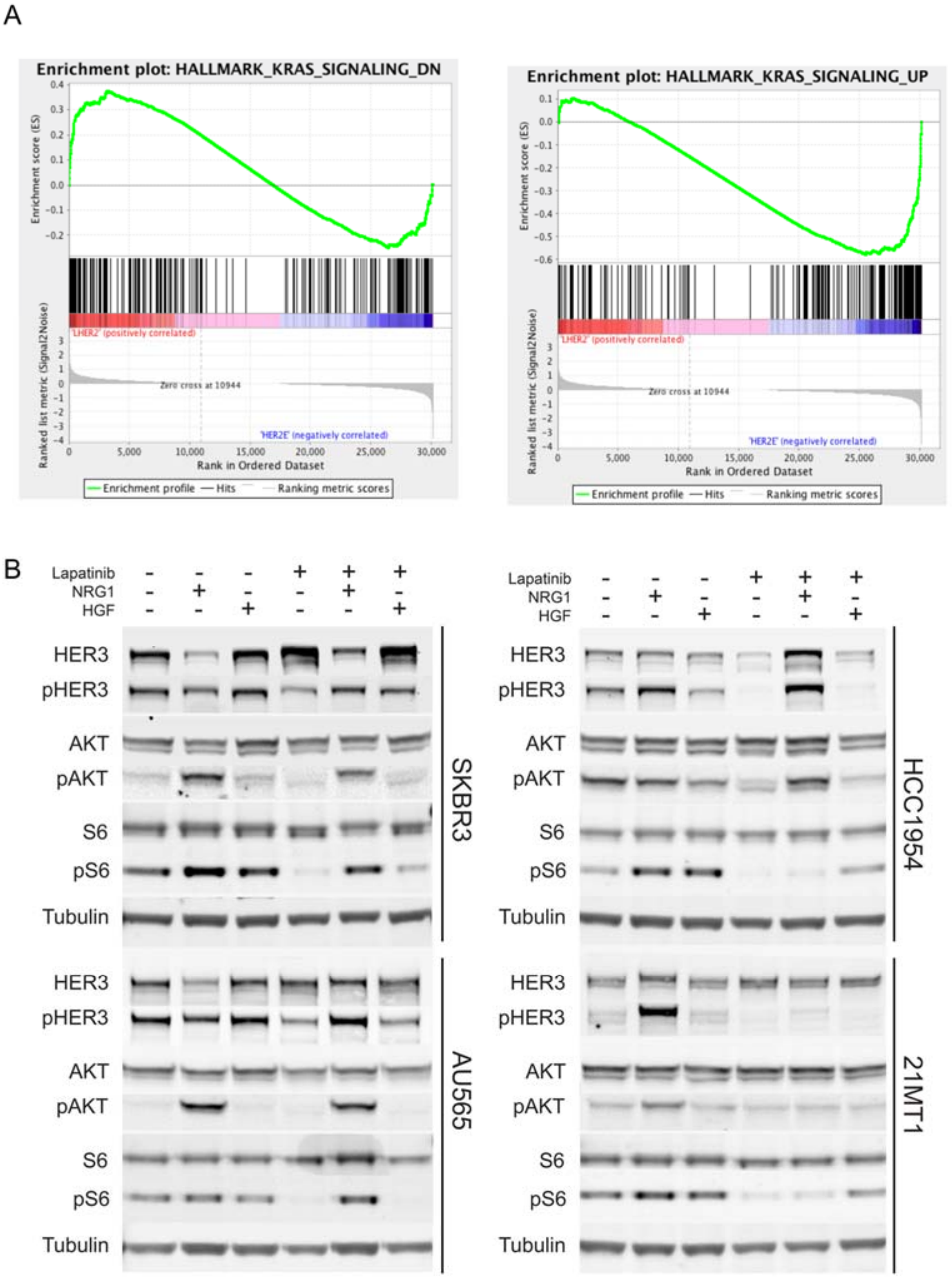

Supplemental Figure S5. Related to Figure 4. L-HER2+ and HER2E have differential MAPK pathway activity and response to HER2 inhibition. (**a**) GSEA comparison plots of L-HER2+ (n = 8) and HER2E lines (n = 8). Top plot shows Hallmark gene set KRAS Down enrichment for L-HER2 vs HER2E (NOM p-val = .003, FDR q-val = .098), and bottom shows KRAS Up gene set enrichment for the reverse comparison (NOM p-val = 0, FDR q-val = 0). (**b**) Western blot protein analysis of AU565, SKBR3, HCC1954, and 21MT1 cell lines treated for 48 hours with combinations of DMSO, 500 nM lapatinib or neratinib, and 50 ng/ml NRG1β or HGF (BR = 3). 21MT1 cells were treated with neratinib due to innate lapatinib resistance.

Supplemental Figure S6

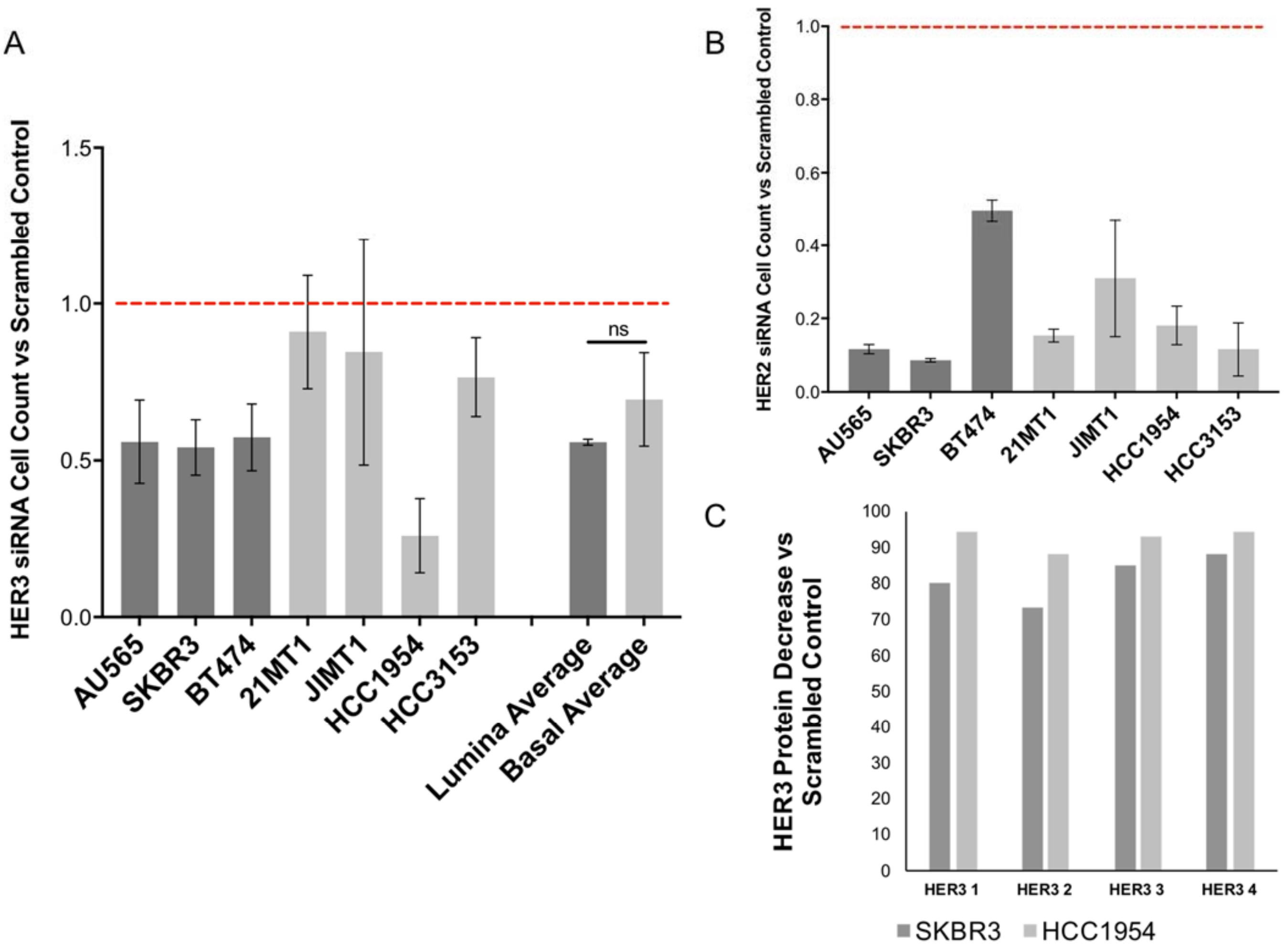

Supplemental Figure S6. Related to Figure 4. HER2E cell lines are less sensitive to HER3 siRNA knockdown than L-HER2+ lines. (**a**) Ratio of mean cell count and SEM (n = 3) of cell lines following 96 hours of HER3 knockdown (averaged over 4 independent HER3 siRNA oligos), normalized to cohorts treated with scrambled siRNA control. Red line indicates equivalent cell counts compared to control. (**b**) Mean cell count and SEM (n = 3) of cell lines following 96 hours of HER2 knockdown, normalized to cohorts treated with scrambled siRNA control. Red line indicates baseline cell count. (**c**) Quantification of HER3 protein immunoblots showing percent decrease in protein levels in SKBR3 and HCC1954 cell lysate following 48 hours of HER3 siRNA knockdown compared to scrambled siRNA controls (BR = 3).

Supplemental Figure S7

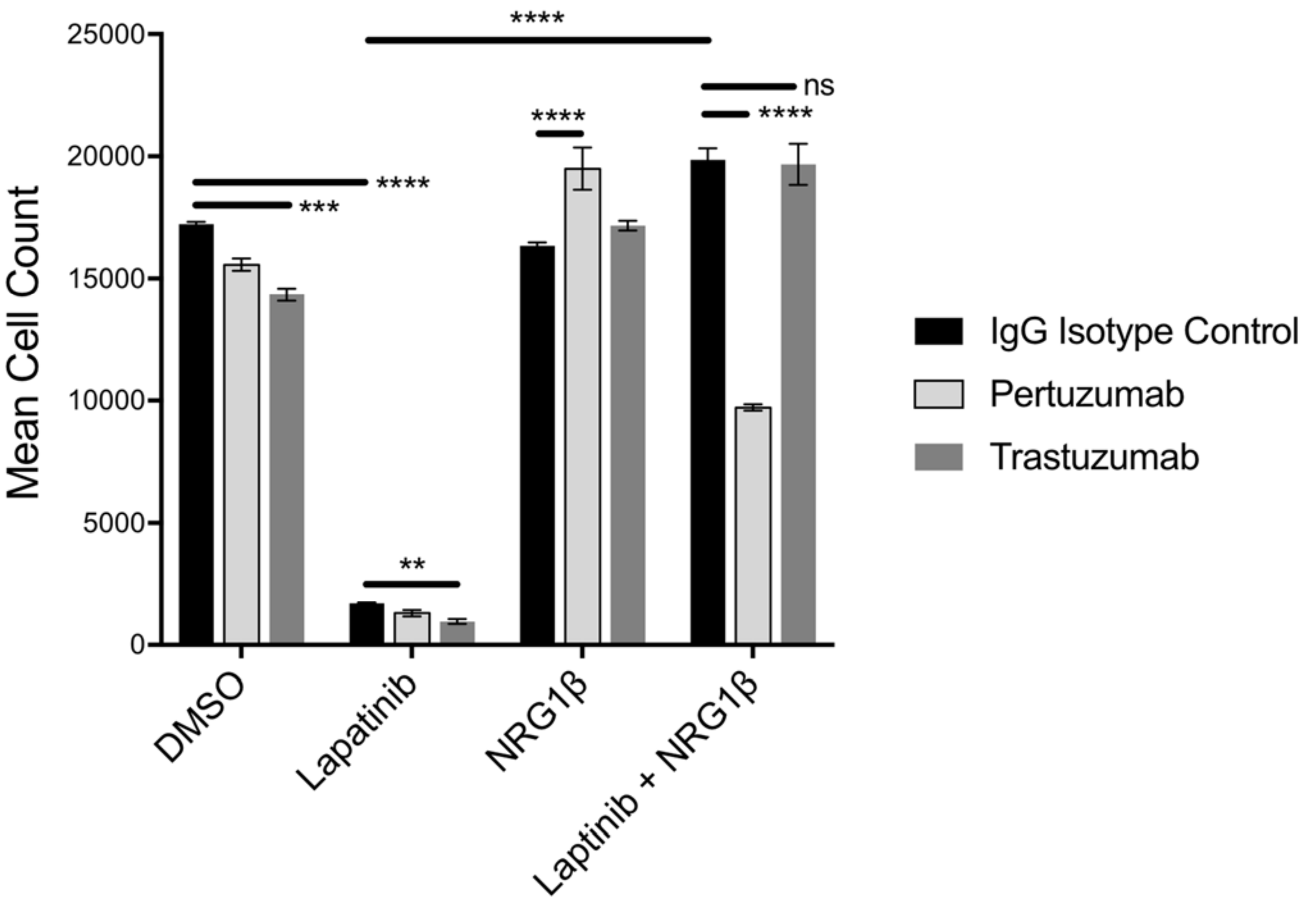

Supplemental Figure S7. Related to Figure 5. Trastuzumab fails to inhibit NRG1β mediated lapatinib resistance. Mean cell count and SEM (n = 3) for AU565 cells treated for 72 hours with combinations of 500 nM lapatinib, 50 ng/mL NRG1β, 30 µg/mL pertuzumab, and 30 µg/mL trastuzumab. PBS, DMSO, and human IgG isotype control were used as controls for growth factors, lapatinib, and pertuzumab or trastuzumb, respectively. Lapatinib significantly decreased cell counts compared to DMSO ($p<0.0001$), and addition of ligand significantly increased cell count compared to lapatinib alone ($p<0.0001$)). Trastuzumab significantly decreased cell count compared to DMSO ($p=0.0005$). The combination of pertuzumab and NRG1β significantly increased cell count compared to NRG1β alone ($p<0.0001$). Addition of pertuzumab to lapatinib plus NRG1β significantly decreased cell counts compared to lapatinib plus NRG1β alone ($p<0.0001$), but addition of trastuzumab to lapatinib plus NRG1β did not.
